# Effect of mutations on binding of ligands to guanine riboswitch probed by free energy perturbation and molecular dynamics simulations

**DOI:** 10.1093/nar/gkz499

**Published:** 2019-06-07

**Authors:** Jianzhong Chen, Xingyu Wang, Laixue Pang, John Z H Zhang, Tong Zhu

**Affiliations:** 1 School of Science, Shandong Jiaotong University, Jinan 250357 China; 2 NYU-ECNU Center for Computational Chemistry at NYU Shanghai, Shanghai 200062, China; 3 Shanghai Engineering Research Center of Molecular Therapeutics & New Drug Development, School of Chemistry and Molecular Engineering, East China Normal University, Shanghai 200062, China

## Abstract

Riboswitches can regulate gene expression by direct and specific interactions with ligands and have recently attracted interest as potential drug targets for antibacterial. In this work, molecular dynamics (MD) simulations, free energy perturbation (FEP) and molecular mechanics generalized Born surface area (MM-GBSA) methods were integrated to probe the effect of mutations on the binding of ligands to guanine riboswitch (GR). The results not only show that binding free energies predicted by FEP and MM-GBSA obtain an excellent correlation, but also indicate that mutations involved in the current study can strengthen the binding affinity of ligands GR. Residue-based free energy decomposition was applied to compute ligand-nucleotide interactions and the results suggest that mutations highly affect interactions of ligands with key nucleotides U22, U51 and C74. Dynamics analyses based on MD trajectories indicate that mutations not only regulate the structural flexibility but also change the internal motion modes of GR, especially for the structures J12, J23 and J31, which implies that the aptamer domain activity of GR is extremely plastic and thus readily tunable by nucleotide mutations. This study is expected to provide useful molecular basis and dynamics information for the understanding of the function of GR and possibility as potential drug targets for antibacterial.

## INTRODUCTION

Riboswitches have been regarded as a new class of genetic control elements founded in the 5′-leader region in multiple bacterial messenger RNAs (mRNA) and play significant roles in modulation of gene expression in bacteria, plants and fungi (1,2). Riboswithches consist of two mutually interacting domains, namely the aptamer domain and the expression platform. The aptamer can directly bind to the metabolite molecules and is responsive to intracellular ligand concentration. The expression platform ensures the structural transformation in response to the changes in the aptamer so as to modulate either ribosome binding or transcription antitermination. Presumably, riboswitches modulate gene expression by an allosteric rearrangement due to binding of small metabolite molecules (3–9). Riboswitches have currently attracted interests as potential drug targets for antibacterial (10).

**Figure 1.**
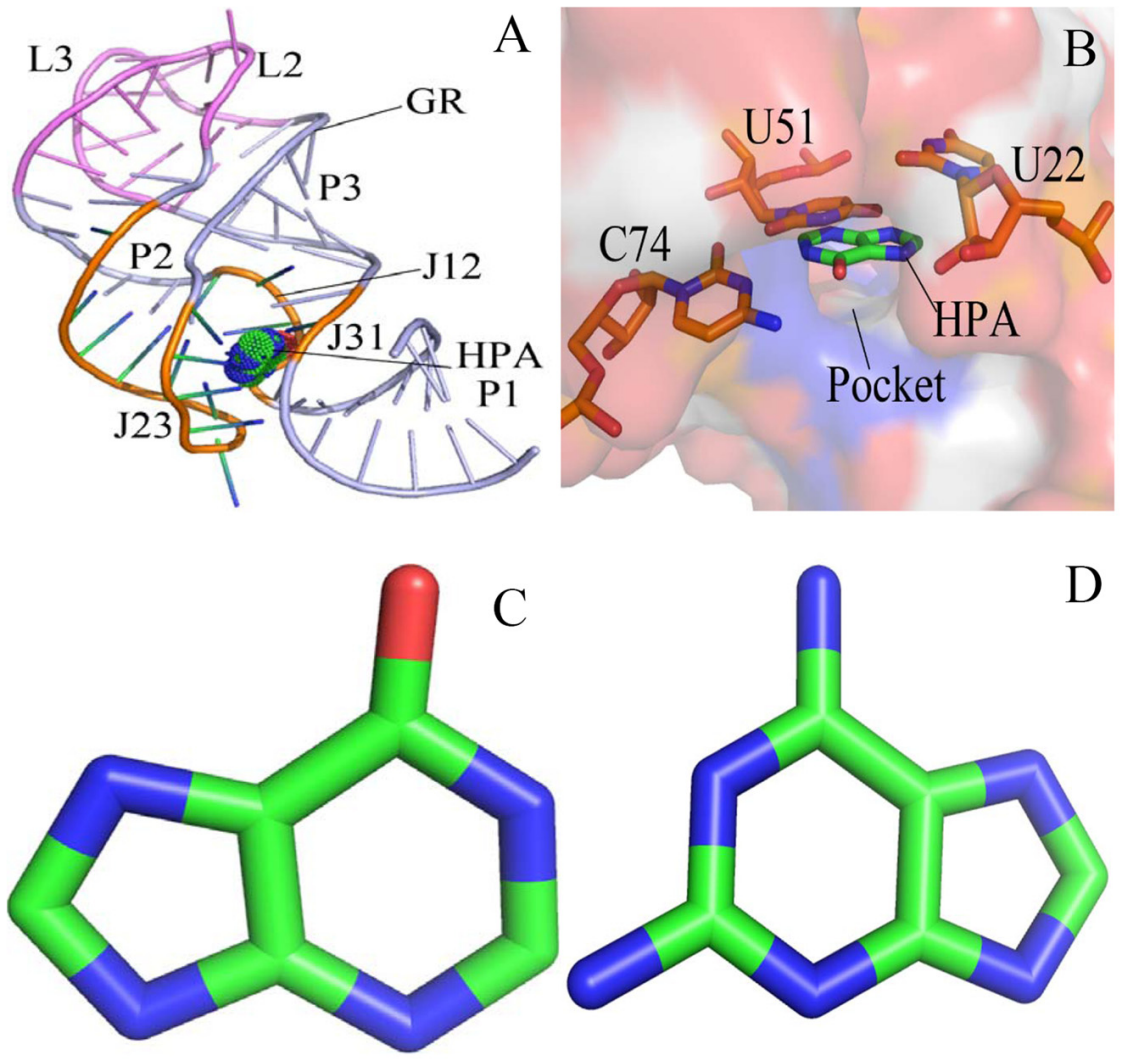
Structures of molecules: (**A**) structure of ligand-GR complex, GR is shown in cartoon modes and ligand in dot modes; (**B**) binding site of ligand to GR, GR is displayed in surface modes and key nucleotides and ligand in stick modes; (**C**) structure of HPA and (D) structure of 6AP. Two ligands HPA and 6AP are depicted in stick modes.

By now, the metabolite molecules recognized by riboswitches mainly include amino acids (11,12), nucleotides (13,14), vitamin cofactors (15,16) and metal ions (17). All riboswitches can not only bind their respective targets with high affinity and selectivity, but also discriminate even against very closely related compounds (18,19). The purine riboswitches, including the guanine-specific riboswitch (GR) and the adenine-sensing riboswitch (AR), were found in 2003 (13,14). The crystallographic structures suggest that these two classes of purine riboswitches consist of three helices P1, P2 and P3 that surround a three-way junction J12, J23 and J31 connecting them, with phylogenetically conserved loops L2 and L3 capping P2 and P3 (20), respectively (Figure 1A and B). The ARs and GRs share highly conserved primary and secondary structure (14), it is observed that a cytosine 74 (C74) is conserved in all GRs and a uridine 74 (U74) is conserved in all ARs. Mutation of C74 into U74 makes the GRs lose the binding ability to guanine and inversely have a strong affinity to adenine (14). Thus, it is of significance to probe molecular mechanism involving the conformational change and ligand binding mechanism of riboswitches for further understanding the role of riboswitches as potential drug targets for antibacterial.

Based on significant roles played by purine riboswitches in the modulation of gene expression, many experimental studies have focused on its structure and function. The previous structural study suggested that the mRNA rearrangement upon ligand binding will build a pocket which can interact with all functional groups of the purine and form a Watson−Crick base pair with C74/U74 of AR/GR (14). This information was further supported by the crystal structures of the ligand-binding domain of the purine riboswitches (20,21). Significantly, the work from Gilbert *et al.* contributed multiple crystal structures of the *Bacillus subtilis xpt-pbuX* GR and its mutant C74U (GR C74U) associated with different ligands and the related binding data (22). Moreover, several groups around the world have performed different experiments to probe the conformational changes of purine riboswitches induced by ligand bindings (23,24). For example, Brenner *et al.* applied single molecule fluorescence resonance energy transfer spectroscopy to explore the conformational changes of the *xpt* GR aptamer (23). Stoddard *et al.* adopted X-ray crystallography and chemical probing to study the effect of modest sequence alterations on the activity of the purine riboswitches, and their results suggested that the introduction of non-natural compositions induces instability to regulate gene expression *in vivo* and the aptamer domain activity is highly plastic and thus readily tunable to meet cellular needs (25). Batey *et al.* performed a detailed mutagenic survey on the *xpt-pbux* GR from *B. subtilis* and the results indicated that the conserved nucleotides, mainly arising from the formation of the ligand-GR complex, promote an open state of the *apo* RNA and take part in the secondary structural switch with the expression platform (19). Therefore, for deeply understanding the role of riboswitches as potential antibacterial drug targets, it is of significance to further investigate the conformational changes of riboswitches and the energy basis of ligand binding to riboswitches.

Currently, MD simulation (26–32) and binding free energy prediction (33–35) have been extensively adopted to explore the interaction mechanism of biomolecules with small organic ligands, such as the ligand-induced conformational change and the free energy basis of the binding. Moreover, MD simulation has been applied to successfully probe the conformational dynamics of RNA influenced by ligand bindings or mutations in sequence (18,36–40). Recently, multiple computational methods with various levels of computational accuracy and expense, including the free energy perturbation (FEP) (41–44), molecular mechanics Poisson−Boltzmann surface area (MM-PBSA) (45–49), and the thermodynamic integration (TI) (50–52) methods, were employed to study the binding affinity of ligands to RNAs. Hu *et al.* combined the TI and MM-PBSA methods to study the binding of four guanine analogues to the GR and GR-C74U, and their results indicated that the loss in binding free energies upon mutation is mainly driven by electrostatic interactions (53). Sund *et al.* integrated the FEP with linear interaction energy (LIE) method to calculate the binding affinities of 14 different purine analogues with GR and AR, and their results demonstrated that the AR specifically uses the electrostatic preorganization to discriminate against guanine by preventing the formation of a G-U wobble base pair (54).

In this study, the influence of sequence mutation on the binding mechanisms of the *xpt-pbuX* GR with ligands are explored by the combination of the MD simulation, FEP, and MM-GBSA method. Two ligands were selected, including hypoxanthine (HPA) and 9H-purine-2,6-diaminopurine (6AP), and their structures were shown in Figure 1C and D. Meanwhile, several mutations of *xpt-pbuX* GR, including A24U, U25A/A46G, A24U/U25A/A46G, AU25A/A46G/C74U, A24U/U25A/A46G/C74U were considered to comparatively probe changes in binding affinities and conformation of the *xpt-pbuX* GR induced by mutations. This study is expected to provide significant energy basis and dynamic information of GRs relating with mutations so as to better understand the role of riboswitches as potential drug targets for antibacterial.

## MATERIALS AND METHODS

### Construction of molecular systems

Atomic coordinates of the wild-type (WT) GR complexed with HPA were obtained from protein data bank (PDB ID: 4FE5) (20). The X-ray structures of HPA complexed with several mutants of GR (A24U, U25A/A46G and A24U/U25A/A46G) can also be obtained from protein data bank (PDB ID: 4FEJ, 4FEL and 4FEN) (25). Atomic coordinates of 6AP complexed with mutated GR (U25A/A46G/C74U and A24U/U25A/A46G/C74U) were taken from protein data bank (PDB ID: 4FEO and 4FEP) (25). The initio structure of 6AP-WT GR complex can be constructed based on the complex structure of HPA with WT GR. The missing hydrogen atoms were connected to the corresponding heavy atoms by using the Leap module in Amber (55,56). The Amber ff99SB force field (57) was employed to produce the topology files of the WT and mutant GRs. The TIP3P model was selected for water molecules. The structures of HPA and 6AP were optimized with the Gaussian 09 program (58) at the HF/6–31G* level, and then the restrained electrostatic potential (RESP) charges and the general Amber force field (GAFF) (59) were assigned to them. The force field parameters of these two ligands are provided as the supplemental data (Files S1–S4). The initialized structures of each complex system was solvated in a truncated octahedral box of TIP3P water molecules with a 14.0 Å buffer along each dimension (60). The appropriate number of sodium counterions were added to neutralize each system. Details of the system preparation were listed in Supplementary Table S1.

### Conventional MD simulations

To remove high energy contacts formed during the initialization of the simulated systems, each system was optimized by energy minimization using a combination of the steepest descent and conjugated gradient methods. Subsequently, each system was gently heated from 0 to 300 K in 1 ns at constant volume and equilibrated at 300 K for another 1 ns. Finally, a 1-μs MD simulation was conducted without any restrictions at constant pressure and 300 K to fully relax each system. In the current work, all MD simulations were ran by using the PMEMD program (61,62) in Amber. During MD simulations, all chemical bonds contain hydrogen atoms were restricted using the SHAKE algorithm (63) and the time step was set to 2 fs. The Langevin thermostat with a collision frequency of 2.0 ps^−1^ was adopted to regulate the temperature of the systems (64). The particle mesh Ewald (PME) (65,66) method was used to compute the long-range electrostatic interactions with the cutoff distances of 12.0 Å, which is also suitable for the calculations of van der Waals interactions. The cross-correlation map and principal component analysis (PCA) (67–71) were carried out using the CPPTRAJ module (72) in Amber and the details involving these analyses have been presented in our previous studies (73,74). Meanwhile, the cluster analysis was also performed using the iMWK-Means method proposed by Salsbury, Jr *et al.* to reveal conformational changes of GRs induced by mutations (67,75,76). The PyMOL (http://www.pymol.org.) and VMD (77) softwares were utilized to check MD trajectories and draw pictures.

### Free energy perturbation calculations

**Figure 2.**
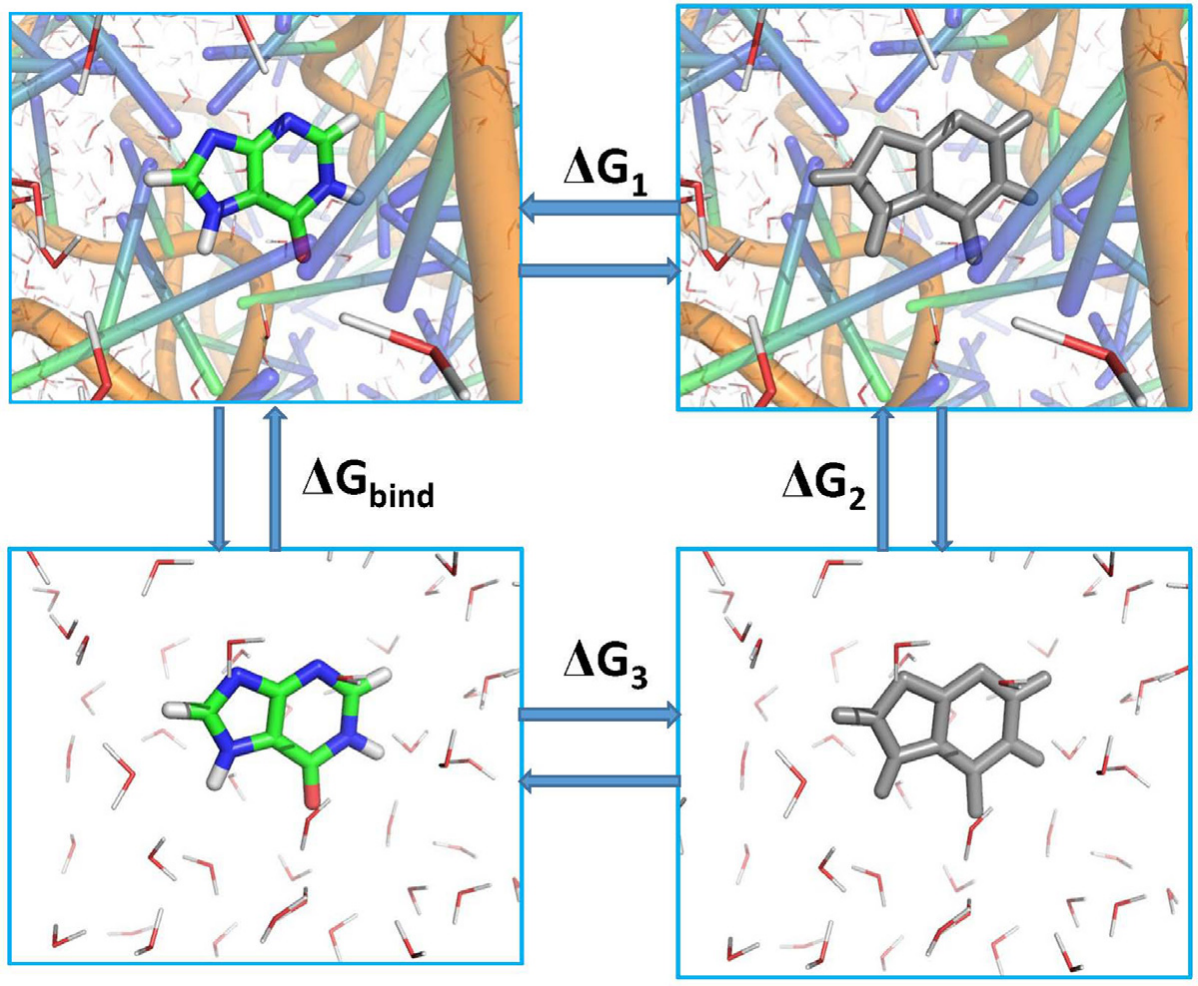
Thermodynamic cycle for calculation of free energy perturbation: $\Delta {G_1}$ and $\Delta {G_3}$ separately corresponding to the changes of free energies in disappearance of ligands from GRs and water, while $\Delta {\rm{\ }}{G_2} = \ 0$. GR is shown in cartoon modes and ligand in stick modes. The color ligand indicates a real molecule and the gray ligand represents a pseudo-molecule without any molecular properties. The red and line molecules indicate water molecules that reflect solvent environment.

The free energy perturbation (FEP) method proposed by Shirts *et al.* (78) was adopted to calculate the absolute binding free energies ($\Delta {G_{bind}}$) of ligands to the WT and mutated GRs. The thermodynamics cycle for the FEP was shown in Figure 2 and $\Delta {G_{bind}}$ can be determined by the following equation

(1) \begin{equation*}\Delta \,{G_{bind}} = \mathop \smallint \nolimits_0^1 {\left\langle {\frac{{\partial V\left( \lambda \right)}}{{\partial \lambda }}} \right\rangle _\lambda }\ d\lambda \end{equation*}

where 1 and 0 indicate the states of the real ligands and the dummy ones without any molecular properties, respectively. λ describes the alchemical coupling parameters transforming from 0 to 1 and ${\langle \ldots \rangle _\lambda }$ denotes an ensemble average in states λ. $\partial V( \lambda )/\partial \lambda$is the derivative of the hydrid potential function on λ. As $\Delta {\rm{\ }}{G_2} = \ 0$, $\Delta {G_{bind}}$ can be represented as

(2) \begin{equation*}\Delta \,{G_{bind}} = \Delta {G_1} - \Delta {G_3}\end{equation*}

in which $\Delta {G_1}$ and $\Delta {G_3}$ represent the change of free energies associate with the disappearance of ligand from GR and bulk water, respectively. In the current study, all FEP calculations were performed by using Gromacs 5.0.4 (79). The last snapshot taken from the first 200 ns of the conventional MD simulation of each system was used as the starting conformation of FEP calculation. The van der Waals and electrostatic interactions between ligands and GRs were annihilated by using a linear alchemical pathway with a λ-value interval of 0.05 for van der Waals interactions, and a λ-value interval of 0.1 for electrostatic interactions (42). In addition, 12 nonuniformly distributed λ values (0.0, 0.01, 0.025, 0.05, 0.075, 0.1, 0.15, 0.2, 0.3, 0.5, 0.75, 1.0) were employed to add restrictions on ligands. In this study, a total of 42 and 31 windows were used for simulations of the ligand-GR complexes and ligands in the bulk water, respectively. For each window, energy minimization of 3000 steps was conducted to remove high-energy contacts between atoms by using the steepest descent algorithm. Subsequently, each system was relaxed for 1ns in the canonical ensemble and the non-hydrogen atoms of the solute were restricted with a force constant of 1000 kJ·mol^−1^·nm^−2^. The temperature in the simulation was controlled at 300K by using a V-rescale thermostat with a response time of 1.0 ps (80). Meanwhile, the pressure of the simulated system was regulated by using the Parrinello-Rahman coupling algorithm with a target pressure of 1 atm (81). For each window, the Berendsen weak coupling algorithm was applied to deeply relax the system for 1 ns in the isothermal-isobaric ensemble in each window (82). Finally, 5 ns production simulation was performed without any restrictions for each window to collect data using Hamiltonian-exchange Langevin dynamics in the *NPT* ensemble. 1 million swaps between the state pair in an interval of 4 ps were carried out by using the Gibbs sampling scheme proposed by Shirts *et al.* (78,83). During simulations, the LINS method (84) was adopted to constrain the chemical bond that contain hydrogen atoms, and the time step of simulation was set to 2 fs. The electrostatic interactions were treated by using the Particle Mesh Ewald (PME) method with a cut-off distance of 12 Å, a spline order of 6, a relative tolerance of 10^−6^ and a Fourier spacing of 1.0 Å. The van der Waals interactions were computed using the soft-core potential and a switch function between 10 and 12 Å (85). The above scheme treating non-bonded interactions aim to relieve singularities, numerical instabilities, and additional minima in the potential energy for all combinations of non-bonded interactions in all intermediate alchemical states (86). Based on the success of FEP method in predicting binding ability of ligands to receptors (42,43,52,87–89), thus FEP method was employed to evaluate the effect of several mutations on binding ability of HPA and 6AP to GRs.

### MM-GBSA calculations

For in-depth comparison, the molecular mechanics generalized Born surface area (MM-GBSA) method (49,90,91) has also been applied to compute the binding free energies of HPA and 6AP to GRs:

(3) \begin{equation*}\Delta \,{G_{bind}} = \Delta {E_{vdW}} + \Delta {E_{ele}} - T\Delta S + \Delta {G_{egb}} + \Delta {G_{esurf}} \end{equation*}

in which the first three terms represent binding free energy components in gas phase and the last two terms denote solvation free energies. $\Delta {E_{vdW}}$and $\Delta {E_{ele}}$ are van der Waals and electrostatic interactions between ligands and GRs and these two components were calculated by using molecular mechanics and ff99SB force field. $ - {{T}}\Delta {{S}}$ indicates the contributions of the entropy changes to ligand associations with GR, which is computed by using the *mmpbsa_py_nabnmode* program (92). $\Delta {G_{egb}}$ is polar solvation energy and can be calculated with the GB model developed by Onufriev *et al.* (93). The last term $\Delta {G_{esurf}}$ is non-polar solvation free energy, which is determined using the following equation

(4) \begin{equation*}\Delta \,{G_{esurf}} = \gamma \times \Delta SASA + \beta \end{equation*}

where the parameter ${\rm{\gamma }}$ and $\Delta SASA$ respectively denote the surface tension and the difference in the solvent accessible surface areas caused by ligand associations. The parameter ${\rm{\beta }}$ reflects a regression offset of the linear relationship. For our current work, ${\rm{\gamma }}$ and ${\rm{\beta \ }}$were assigned as 0.0072 kcal·mol·Å^−2^ and 0 kcal·mol^−1^, respectively (94). Due to the high cost in calculation of the entropy, only 50 snapshots extracted from the equilibrated trajectories were adopted for the entropy calculation, while for calculations of the other free energy components, the total of 200 conformations taken from the equilibrated MD trajectories were used.

## RESULTS

### Effect of mutations on binding ability of ligands to GR

To comparatively evaluate the effect of mutations on the binding ability of ligands to GRs, FEP and MM-GBSA methods were applied to compute the binding affinity of ligands 6AP and HPA to the WT and mutated GRs. The calculated results from FEP and MM-GBSA methods are independently listed in Table 1 and 2. Due to the lack of experimental free energy information involving mutations on the ligand-GR bindings, the correlation relationship between binding free energies computed by FEP method and MM-GBSA method is estimated to evaluate the reliability and the results are depicted in Supplementary Figure S1. It is observed that the correlation coefficient (*R*^2^) reaches 0.83, which suggests that our current free energy calculations on predicting the binding affinity of HPA and 6AP to the WT and mutated GRs are reliable.

**Table 1.** Binding free energies of HPA and 6AP to the WT and mutated GRs calculated by using free energy perturbation method^a^

Ligands	RNA	^b^ $\Delta {G_3}$	^b^ $\Delta {G_1}$	$\Delta {G_{bind}}$	^c^ $\Delta \Delta {\rm{G}}$
HPA	WT	–20.86±0.12	–27.78±0.23	–6.92±0.18	
	A24U	–20.86±0.12	–28.55±0.13	–7.69±0.13	–0.77
	U25A/A46G	–20.86±0.12	–28.31±0.43	–7.45±0.23	–0.53
	A24U/U25A/A46G	–20.86±0.12	–28.94±0.34	–8.08±0.23	–1.16
6AP	WT	–21.09±0.14	–33.46±0.47	–12.37±0.31	
	U25A/A46G/C74U	–21.09±0.14	–36.55±0.58	–15.46±0.36	–3.09
	A24U/U25A/A46G/C74U	–21.09±0.14	–34.60±0.36	–13.51±0.25	–1.14

^a^All values are in kcal/mol.

^b^ $\Delta {G_1}$and $\Delta {G_3}$ represents the changes of free energies in decoupling ligands from GRs and water, respectively, $\Delta {\rm{\ }}{G_{bind}} = \ \Delta {G_1} - \Delta {G_3}$.

^c^ $\Delta \Delta {\rm{G\ }} = {\rm{\ }}\Delta {G^{mutant}} - \Delta {G^{wild}}$.

**Table 2.** Binding free energies and separate free energy components of HPA and 6AP to the WT and mutated GRs calculated by using MM-GBSA method^a^

Ligands	GRs	^d^ $\Delta {E_{vdW}}$	$\Delta {E_{ele}}$	$\Delta {G_{egb}}$	$\Delta {G_{esurf}}$	^b^ $\Delta {G_{ele + egb}}$	^e^ $\Delta {\rm{H}}$	$ - {\rm{T}}\Delta S$	$\Delta {G_{bind}}$	$\Delta \Delta {\rm{G}}$
HPA	WT	–26.13±0.38	–21.27±1.05	32.30±0.75	–2.06±0.02	11.03±0.80	–17.16±0.55	15.02±0.56	–2.14	
	A24U	–23.73±0.46	–22.79±0.63	30.47±0.60	–2.02±0.04	7.68±0.62	–18.07±0.43	14.87±0.66	–3.30	–1.06
	U25A/A46G	–28.53±0.23	–8.78±0.34	22.41±0.36	–2.22±0.08	13.63±0.35	–17.12±0.26	13.33±0.62	–3.79	–1.65
	A24U/U25A/A46G	–25.40±0.18	–31.03±0.37	35.27±0.25	–2.24±0.01	4.24±0.31	–23.40±0.21	15.82±0.99	–7.58	–5.44
6AP	WT	–25.56±0.27	–36.47±0.30	37.12±0.23	–2.27±0.01	0.65±0.21	–27.18±0.22	19.82±0.68	–7.36	
	U25A/A46G/C74U	–24.75±0.31	–38.88±0.53	35.53±0.44	–2.28±0.01	–3.35±0.49	–30.38±0.34	18.41±0.61	–11.97	–4.61
	A24U/U25A/A46G/C74U	–27.12±0.21	–35.98±0.41	35.09±0.38	–2.34±0.01	–0.89±0.38	–30.35±0.26	19.10±0.88	–11.25	–3.89

^a^All values are in kcal/mol, ^b^$\Delta {\rm{\ }}{G_{ele + egb}} = {\rm{\ }}\Delta {E_{ele}} + \Delta {G_{egb}}$, ^c^$\Delta \Delta {\rm{G\ }} = {\rm{\ }}\Delta {G^{mutant}} - \Delta {G^{wild}}$, ^d^The symbol ‘±’ indicates the standard errors of mean, ^e^$\Delta {\rm{H\ }} = {\rm{\ }}\Delta {E_{vdW}} + \Delta {E_{ele}} + \Delta {G_{egb}} + \Delta {G_{surf}}$.

In FEP calculations, 5-ns production simulations were performed on each window to obtain fully statistical samplings and the last 3-ns trajectories of each window simulation were utilized to estimate the binding free energies by using the *g_bar* tool in Gromacs 5.0.4 (Table 1). In Table 1, $\Delta {G_3}$and $\Delta {G_1}$ represent the changes of free energies in decoupling ligands from water and GRs, respectively. As shown in Table 1, the change of free energy caused by decoupling HPA from water is close to that caused by decoupling 6AP from water and the difference between them is only 0.23 kcal/mol. However, the free energy change induced by decoupling 6AP from the binding site of the WT GR is 5.68 kcal/mol stronger than that caused by disappearing HPA from the WT GR, which provides significant contribution to the difference in the binding ability of HPA and 6AP to the WT GR. According to Table 1, the binding affinity of 6AP to the WT GR is strengthened by 5.45 kcal/mol relative to that of HPA to the WT GR, suggesting that binding strength of 6AP to the WT GR is much stronger than HPA and also implying that the WT GR has a binding preference and selectivity for 6AP over HPA. Comparing to the WT GR, the mutations A24U, U25A/A46G and A24U/U25A/A46G separately lead to the strengthening of 0.77, 0.53 and 1.16 kcal/mol in binding abilities of HPA to GR. Similarly, the mutations U25A/A46G/C74U and A24U/U25A/A46G/C74U respectively result in the enhancing of 3.09 and 1.14 kcal/mol in the binding affinity of 6AP to the mutated GR relative to the WT one. Based on the above results, it is concluded that the GR mutants involved in the current work obviously strengthen the binding ability of ligands HPA and 6AP to the mutated GRs.

In MM-GBSA calculations, 1-μs MD simulations were performed on the seven studied systems. Root-mean-square deviations (RMSD) of atoms P, O3′, O5′ C3′, C4′ and C5′ in GR were computed relative to the first structure through the entire MD simulations to evaluate the reliability of system equilibrium and the results were displayed in the supporting information (Supplementary Figure S2). It is observed that all systems basically reach the equilibrium after 200 ns of MD simulations. To obtain a good conformational sampling, a total of 200 conformations extracted from the last 800 ns trajectories of MD simulations with a time interval of 4 ns were used to compute the binding free energies. Binding free energies and separate free energy components were listed in Table 2.

According to Table 2, binding free energies are decomposed into van der Waals interactions ($\Delta {E_{vdW}}$), electrostatics interactions ($\Delta {E_{ele}}$), entropy contributions ($ - T\Delta S$), polar solvation free energies ($\Delta {G_{egb}}$) and non-polar solvation free energies ($\Delta {G_{esurf}}$). It is found that the binding free energy of 6AP to the WT GR is strengthened by 5.22 kcal/mol comparing to that of HPA to the WT GR, indicating that the binding ability of 6AP to the WT GR is stronger than HPA. Meanwhile, comparing to the WT GR, binding free energies of HPA to the mutated GRs are separately enhanced by 1.16, 1.65 and 5.44 kcal/mol due to the mutations A24U, U25A/A46G and A24U/U25A/A46G, while that of 6AP to the mutated GRs are improved by 4.61 and 3.89 kcal/mol because of the mutations U25A/A46G/C74U and A24U/U25A/A46G/C74U, suggesting that these mutations strengthen the binding of two ligands to the mutated GRs. These results agree well with the previous FEP calculations. As shown in Table 2, although A24U and A24U/U25A/A46G lead to the decrease of 2.40 and 0.73 kcal/mol in van der Waals interactions of HPA with GR, the polar interactions of HPA with GR ($\Delta {G_{ele + egb}}$), an unfavorable factor generated by the sum of electrostatic interactions and polar solvation free energies, are decreased by 3.35 and 6.79 kcal/mol in the two mutated systems, which completely compensate the decrease in van der Waals interactions. Thus, the decrease in polar interactions of HPA with GR induced by A24U and A24U/U25A/A46G is a main force of enhancing the binding ability of HPA to the mutated GRs. According to Table 2, comparing to the WT GR, the increase of 2.60 kcal/mol in the polar interaction of HPA with the U25A/A46G mutated GR weakens the binding of HPA to GR, but the van der Waals interaction of HPA with the U25A/A46G mutated GR is improved by 2.40 kcal/mol, besides the entropy change ($ - {\rm{T}}\Delta S$) due to the mutations U25A/A46G is also decreased by 1.69 kcal/mol relative to the WT one. Thus, it totally strengthens the binding ability of HPA to the U25A/A46G mutated GR. In the case of 6AP, mutations U25A/A46G/C74U generate a decrease of 0.81 kcal/mol in van der Waals interactions of 6AP with the mutated GR relative to the WT one, which slightly impairs the association of 6AP with GR. However, the mutations U25A/A46G/C74U respectively lead to the decrease of 4.00 kcal/mol and 1.41 kcal/mol in polar interactions and entropy changes compared to the WT GR, which not only completely compensate the decrease in van der Waals interactions of 6AP with the U25A/A46G/C74U mutated GR, but also totally strengthen the binding of 6AP to the mutated GR. As seen in Table 2, the decrease of 0.72 kcal/mol in the entropy changes induced by the mutations A24U/U25A/A46G/C74U softly strengthens the association of 6AP with the mutated GR. However A24U/U25A/A46G/C74U produces the decrease of 1.56 kcal/mol in van der Waals interaction and 1.54 kcal/mol in polar interaction of 6AP with GR, which totally enhances binding of 6AP to the A24U/U25A/A46G/C74U mutated GR. Based on the above analysis of the binding free energies from FEP and MM-GBSA calculations, the mutations mentioned in this study enhance the bindings of HPA and 6AP to GRs, implying that these mutations may exert a dramatic impact on the activity of GR. The work from Stoddard *et al.* also suggested that the aptamer domain activity of GR is highly plastic and thus readily tunable to meet cellular needs, which is in good agreement with our current conclusions (25).

### Impact of mutations on ligand-GR interaction networks

**Figure 3.**
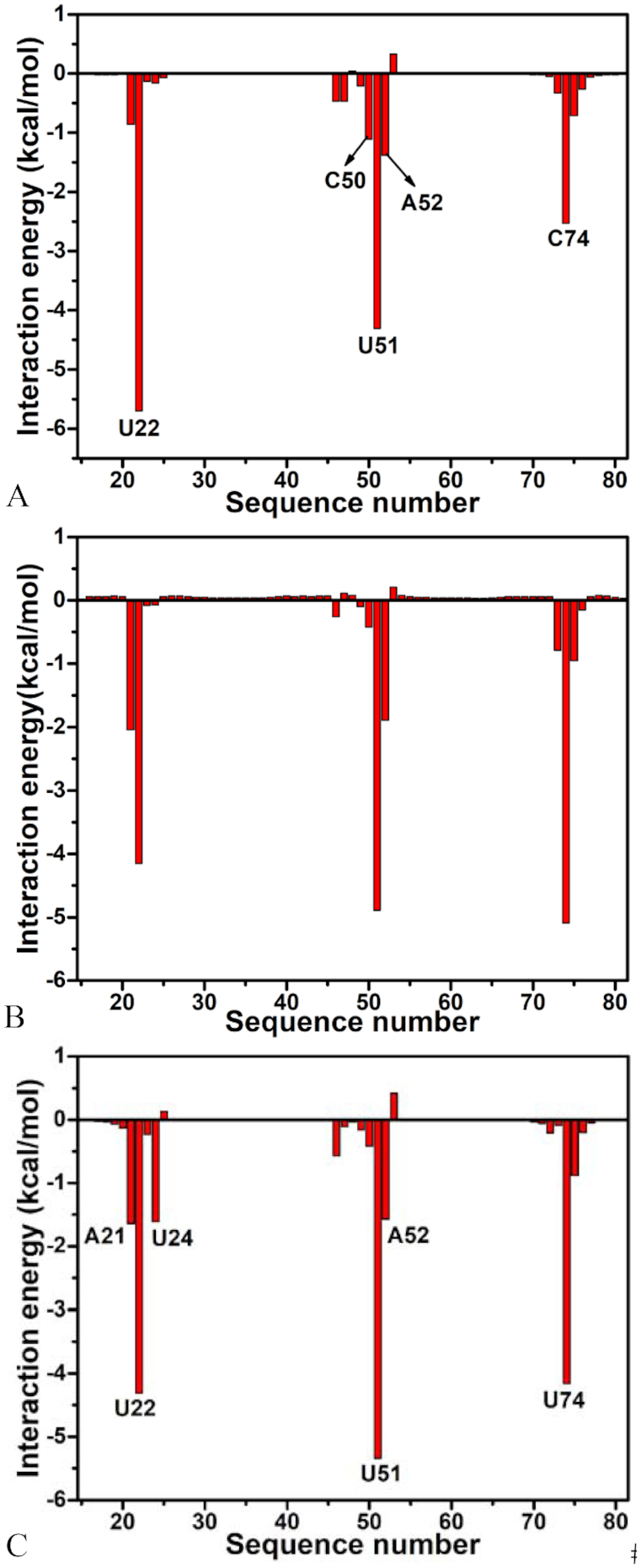
6AP-nucleotide interaction spectrum: (**A**) the WT GR; (**B**) the GR with mutations U25A/A46G/C74U and (**C**) the GR with mutations A24U/U25A/A46G/C74U. Only nucleotides of interaction energies stronger than 1.0 kcal/mol are labeled.

**Figure 4.**
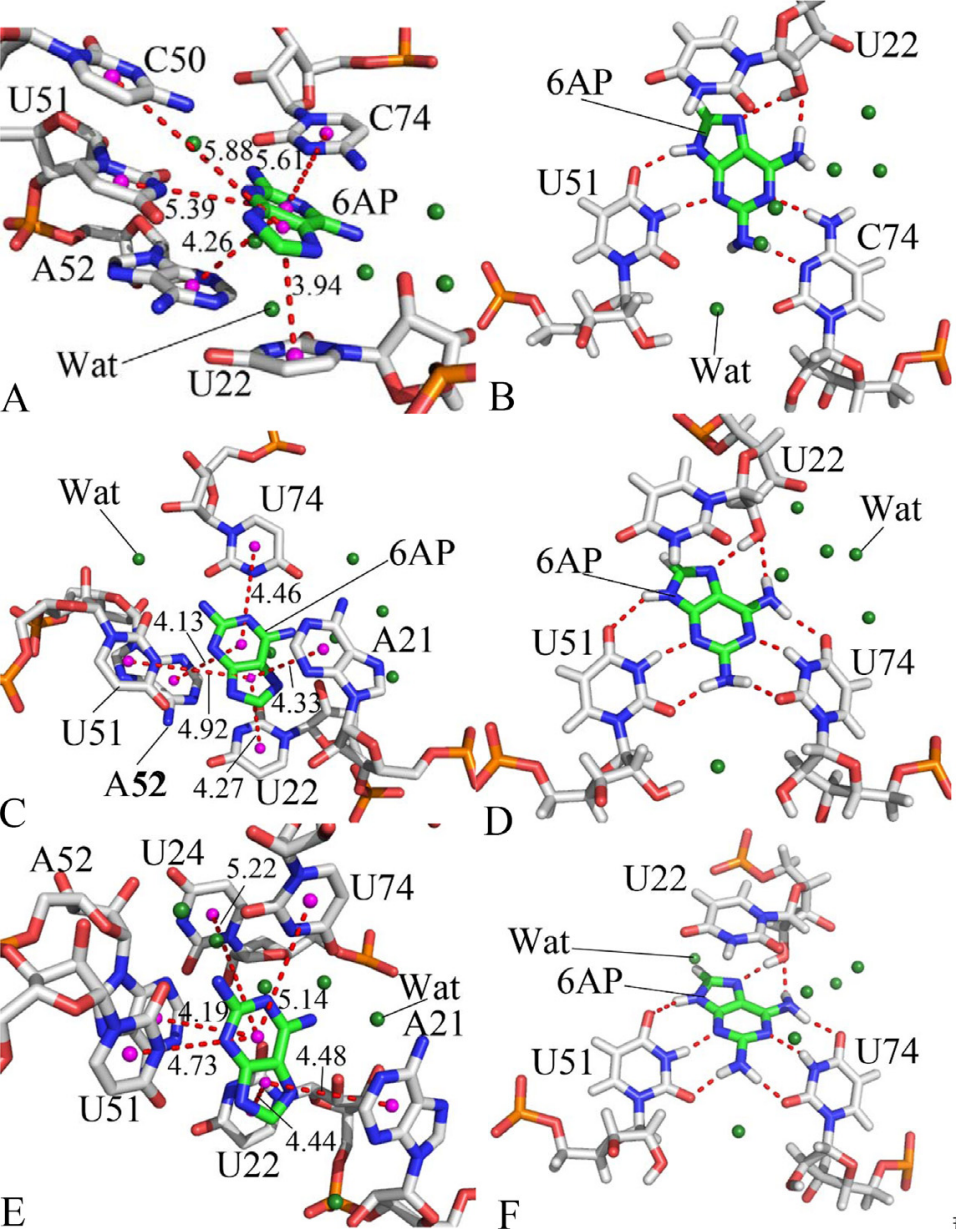
Significant interactions of 6AP with key nucleotides in GR: (**A**) and (**B**) respectively indicating hydrophobic interactions and hydrogen bonding interactions of 6AP with nucleotides in the WT GR; (**C**) and (**D**) separately depicting hydrophobic interactions and hydrogen bonding interactions of 6AP with nucleotides in GR with mutations U25A/A46G/C74U; (**E**) and (**F**) respectively describing hydrophobic interactions and hydrogen bonding interactions of 6AP with nucleotides in GR with mutations A24U/U25A/A46G/C74U. The key nucleotides and 6AP are shown in stick modes and water molecules in the forest color balls. The magenta balls represent pseudoatoms forming hydrophobic interactions between 6AP and nucleotides.

**Figure 5.**
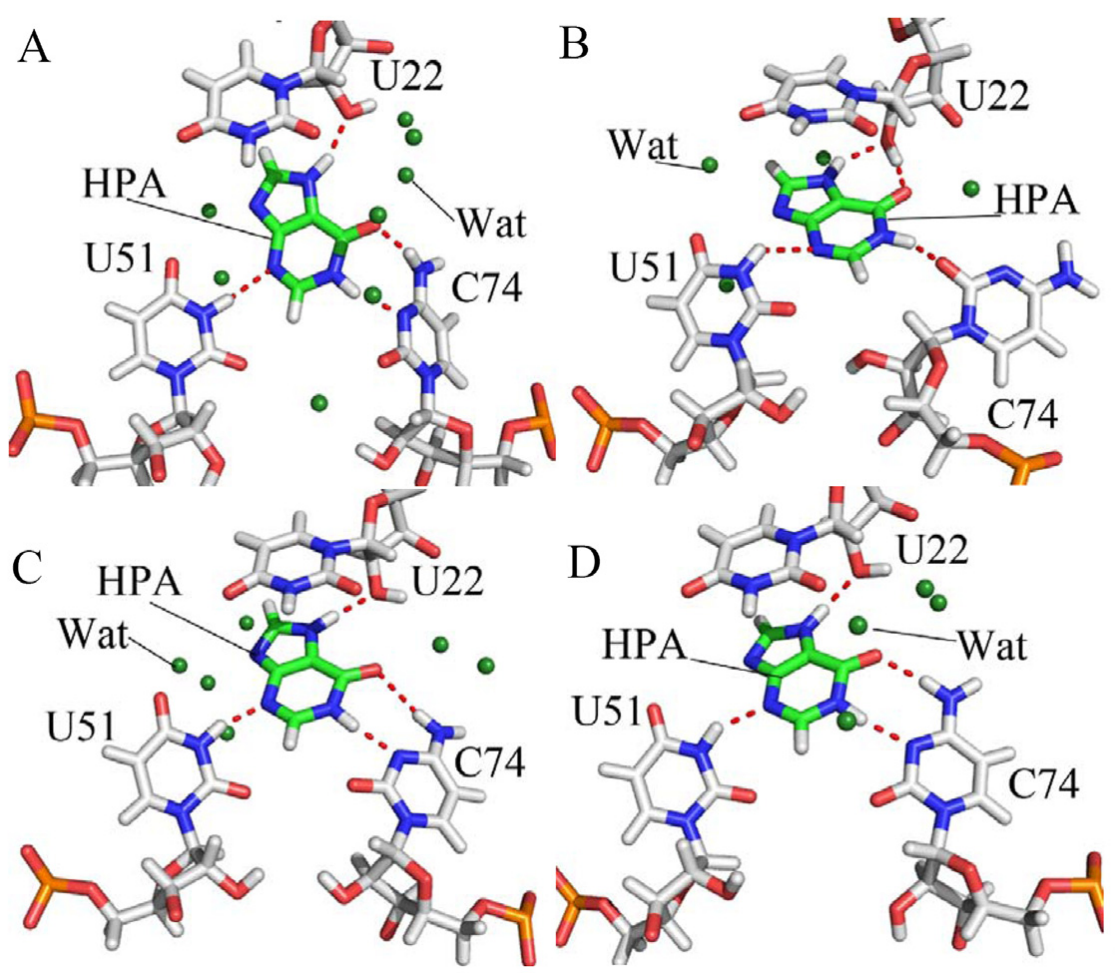
Hydrogen bonding interactions of HPA with key nucleotides in the WT and mutated GRs: (**A**) WT; (**B**) A24U; (**C**) U25A/A46G and (**D**) A24U/U25A/A46G. The key nucleotides and HPA are shown in stick modes and water molecules in the forest color balls.

To reveal the molecular mechanism under the strengthened binding ability of HPA and 6AP to mutant GRs, residue-based free energy decomposition method was utilized to compute the interaction spectrum of 6AP and HPA with the separate nucleotides of GRs (Figure 3 and Supplementary Figure S3). Geometrical positions of 6AP and HPA relative to the key nucleotides in GRs were depicted in Figures 4, 5 and Supplementary Figure S4 by using the averaged structures taken from the last 10 ns of MD trajectories. Hydrogen bonding interactions of 6AP and HPA with nucleotides were analyzed with the program CPPTRAJ in Amber and the information was listed Table 3 and Supplementary Table S2.

**Table 3.** Hydrogen bonding interactions of ligand 6AP with key nucleotides in WT and mutated GR

	WT GR		U25A/A46G/C74U		A24U/U25A/A46G/C74U	
^a^Hydrogen bonds	^b^Occu (%)	Dista (Å)	Occu (%)	Dista (Å)	Occu (%)	Dista (Å)
^c^6AP@N3…U51@N3-H3	95.24	2.92	98.65	2.89	99.41	2.91
6AP@N1…C74@N4-H4	93.26	2.99	–	–	–	–
6AP@N7…U22@O2′-HO2′	85.68	2.82	98.44	2.81	94.12	2.82
U51@O4…6AP@N9-H1	74.41	3.10	78.62	3.06	79.8	3.01
C74@N3…6AP@N2-H2	62.70	3.01	–	–	–	–
U22@O2′…6AP@N6-H6	41.11	3.02	28.87	3.07	55.86	3.01
6AP@N1…U74@N3-H3	^d^–	–	99.58	2.93	98.22	2.95
U74@O4…6AP@N6-H5	–	–	94.22	2.91	88.53	2.96
U74@O2…6AP@N2-H4	–	–	97.09	2.88	95.71	2.91
U51@O2…6AP@N2-H3	–	–	96.38	2.90	88.64	2.95

^a^Hydrogen bonds are determined by the acceptor$ \cdots$donor distance of <3.5Å and acceptor$ \cdots$H-donor angle of >120°.

^b^Occu (%) represent occupancy of hydrogen bonds that is defined as the percentage of simulation time that a specific hydrogen bond exists.

^c^The full lines indicate chemical bonds, and the dotted lines describe hydrogen bonding interactions.

^d^The symbol ‘–’ indicates that the occupancy of hydrogen bonds formed between 6AP and nucleotides is lower than 20%.

As shown in Figure 3A, ligand 6AP produces interactions stronger than 1.0 kcal/mol with five nucleotides in the WT GR, including U22, C50, U51, A52 and C74. Among these nucleotides, the interaction of 6AP with U22 is the strongest, which provides an energy contribution of -5.70 kcal/mol for the binding of 6AP to the WT GR. Structurally, this interaction mainly arises from the π-π stack interaction of the hydrophobic ring of 6AP with that of U22 (Figure 4A) and hydrogen bonding interactions between 6AP and U22 (Figure 4B), involving two hydrogen bonds 6AP@N7…U22@O2′-HO2′ and U22@O2′…6AP@N6-H6 with the occupancy of 85.68% and 41.11%, respectively (Table 3). Similar to U22, nucleotides U51 and C74 also produce the π-π stack interactions and hydrogen bonding interactions with 6AP, which separately contribute interaction energies of –4.31 and –2.53 kcal/mol to the association of 6AP with the WT GR. The hydrogen bonding interactions of these two nucleotides with 6AP mainly include 6AP@N3…U51@N3-H3, U51@O4…6AP@N9-H, 6AP@N1…C74@N4-H4 and C74@N3…6AP@N2-H2 and their occupancy are 95.24%, 74.41%, 93.26% and 62.70%, respectively (Figure 4B and Table 3), indicating that these hydrogen bonds are stable during MD simulations. The interaction energies of 6AP with C50 and A52 are –1.11 and –1.38 kcal/mol, separately, which structurally agree with the π–π stack interactions of the hydrophobic ring of 6AP with that of C50 and A52. Comparing to the WT GR, mutations U25A/A46G/C74U induce the changes of the 6AP-nucleotide interactions (Figure 3B and Table 3). Although the interactions of 6AP with U22 and C50 are weakened by 1.55 and 0.69 kcal/mol due to mutations U25A/A46G/C74U compared to the WT GR, respectively, that of 6AP with U51, A52 and U74 are separately strengthened by 0.58, 0.51 and 2.56 kcal/mol relative to the WT GR. Structurally, the hydrophobic ring of A52 generates the π-π stack interactions with that of 6AP (Figure 4C), and this interaction is enhanced by 0.51 kcal/mol due to mutations U25A/A46G/C74U compared to the WT GR. According to Table 3, despite of the disappearance of two hydrogen bonds 6AP@N1…C74@N4-H4 and C74@N3…6AP@N2-H2 in the mutated GR with 6AP, mutations U25A/A46G/C74U induced the formation of four new hydrogen bonds, including 6AP@N1…U74@N3-H3, U74@O4…6AP@N6-H5, U74@O2…6AP@N2-H4 and U51@O2…6AP@N2-H3 with the occupancy of 99.58%, 94.22%, 97.09% and 96.38%, respectively (Figure 4D and Table 3), suggesting that these four hydrogen bonds are very stable during MD simulation. Except for the decrease in the occupancy of U22@O2′…6AP@N6-H6, the occupancy of other three hydrogen bonds 6AP@N3…U51@N3-H3, 6AP@N7…U22@O2′-HO2′ and U51@O4…6AP@N9-H1 are increased caused by mutations U25A/A46G/C74U. In summary, the changes in interacting hydrogen bonds in 6AP-nuleotide interactions caused by U25A/A46G/C74U enhance the binding of 6AP to the mutated GR. Similar to U25A/A46G/C74U, mutations A24U/U25A/A46G/C74U also generate obvious influence on the 6AP-nucleotide interactions (Figure 3C and Table 3). As shown in Figure 3C, despite of the decrease of 1.39 and 0.69 kcal/mol in interactions of 6AP with U22 and C50 caused by mutations A24U/U25A/A46G/C74U, these mutations also lead to the strengthening of 0.78, 1.48, 1.03, 0.19 and 1.63 kcal/mol in the interactions of 6AP with A21, U24, U51, A52 and U74 compared to the WT one (Figure 4E), respectively. Although two hydrogen bonds 6AP@N1…C74@N4-H4 and C74@N3…6AP@N2-H2 lose due to mutations A24U/U25A/A46G/C74U, these mutations induce four new hydrogen bonds 6AP@N1…U74@N3-H3, U74@O4…6AP@N6-H5, U74@O2…6AP@N2-H4 and U51@O2…6AP@N2-H3, separately with the occupancy of 98.22%, 88.53%, 95.71% and 88.64%, implying that these hydrogen bonds are stable through MD simulations. Additionally, the occupancy of other four hydrogen bonds 6AP@N3…U51@N3-H3, 6AP@N7…U22@O2′-HO2′, U22@O2′…6AP@N6-H6 and U51@O4…6AP@N9-H1 are also increased by mutations A24U/U25A/A46G/C74U relative to the WT GR (Table 3 and Figure 4F). Thus, the above changes in the 6AP-nucleotide interactions induced by mutations A24U/U25A/A46G/C74U totally improve the binding ability of 6AP to the mutated GR compared to the WT one.

For ligand HPA, five nucleotides A21, U22, U51, A52 and C74 provide energy contributions stronger than 1.0 kcal/mol for the binding of HPA to the WT GR (Supplementary Figure S3A). Among these nucleotides, the interaction energies of HPA with A21 and A52 are –1.42 and –2.42 kcal/mol, respectively, which mainly originate from the π–π stack interactions of the hydrophobic rings of HPA with that of A21 and A52 (Supplementary Figure S4A). According to Supplementary Table S2 and Figure 5A, HPA forms a hydrogen bonding interaction HPA@N3…U51@N3…H3 with U51 with an occupancy is 58.24%, which contributes an interaction energy of -1.10 kcal/mol to the association of HPA with the WT GR (Supplementary Table S2 and Figure 5A). Structurally, U22 and C74 not only form the π–π stack interaction with HPA (Supplementary Figure S4A), but also produce three hydrogen bonding interactions with HPA, including HPA@O6…C74@N4…H41, U22@O2′…HPA@N7-H3 and C74@N3…HPA@N1-H1 with the occupancy of 73.92%, 80.17% and 78.84% (Supplementary Table S2 and Figure 5A), separately. These two nucleotides respectively provide the energy contributions of –4.13 and –4.50 kcal/mol to the binding of HPA to the WT GR (Supplementary Figure S3A). Comparing to the WT GR, mutation A24U exerts obvious impact on HPA-nucleotide interactions (Supplementary Figure S3B). It is observed that interaction energies of HPA with A21, U22 and C74 are reduced by 0.61, 1.01 and 2.16 kcal/mol compared to the WT GR due to the mutation A24U. However, interaction energies of HPA with U51, A52 and U75 in the A24U mutated GR are strengthened by 0.46, 0.57 and 3.38 kcal/mol relative to the WT one (Supplementary Figures S3 and S4B). Although A24U leads to the disappearance of hydrogen bonds C74@N3…HPA@N1-H1 and HPA@O6…C74@N4…H41, but also induces the formation of two new hydrogen bonds HPA@O6…U22@O2′-HO2′ and C74@O2…HPA@N1-H1 with the occupancy of 33.32% and 98.58% respectively (Figure 5B and Supplementary Table S2). More importantly, the occupancy of other two hydrogen bonds HPA@N3…U51@N3…H3 and U22@O2′…HPA@N7-H3 in the A24U mutated GR is obviously increased relative to the WT one. As a whole, the changes in the HPA-nucleotide interactions induced by A24U strengthen the binding of HPA to the mutated GR. By comparison with the WT GR, except for U51, mutations U25A/A46G also generate obvious effect on HPA-nucleotide interactions. As shown in Supplementary Figure S3C, although the interactions of HPA with U22 and A52 in the U25A/A46G mutated GR are separately weakened by 0.77 and 0.58 kcal/mol, that of HPA with A21, C74 and U75 are strengthened by 0.83, 0.71 and 0.58 kcal/mol due to U25A/A46G mutations (Supplementary Figures S3C and S4C). Apart from the decrease in the occupancy of hydrogen bond HPA@N3…U51@N3…H3, the occupancy of other three hydrogen bonds HPA@O6…C74@N4…H41, U22@O2′…HPA@N7-H3 and C74@N3…HPA@N1-H1 are all increased because of mutations U25A/A46G compared to the WT GR (Supplementary Table S2 and Figure 5C). For mutations A24U/U25A/A46G, the impact of mutations on the HPA-nucleotide interactions is highly apparent (Supplementary Figure S3D). Interaction energies of HPA with U22, A52 and C74 are separately reduced by 0.63, 0.83, and 0.97 kcal/mol, but that of HPA with A21, C50 and U51 are enhanced by 1.53, 1.89, and 0.69 kcal/mol due to mutations A24U/U25A/A46G relative to the WT GR (Supplementary Figures S3D and S4D), respectively. Moreover, it is also noted that the occupancy of all four hydrogen bonds, including HPA@O6…C74@N4…H41, HPA@N3…U51@N3…H3, U22@O2′…HPA@N7-H3 and C74@N3…HPA@N1-H1, are increased by mutations A24U/U25A/A46G compared to the WT GR (Supplementary Table S2 and Figure 5D). Thus, the changes in HPA-nucleotide interactions caused by A24U/U25A/A46G strengthen the association of HPA with the mutated GR.

In addition, it is worth noting that several water molecules were observed in the binding pocket of all averaged structures taken from the last 10 ns trajectories of MD simulations, which indicates that water molecules may be of significance for the binding of ligand to GRs and function of GRs. Consistent with our current results, Sund *et al.* also suggested that several water molecules appear in the averaged structure of GR from MD simulations (54). Meanwhile, Batey *et al.* also found that 5% of the surface in the center of a three-way helical junction is still solvent accessible and a conserved water molecule is observed in the highest-resolution crystal structures to form the bridge interaction between the carbonyl groups of U/C74 and U51 (20,95), which may show an induced fit mechanism of binding. Except for the crystal structure, the molecular-docked structure performed by Daldrop *et al.* also detected the existence of water molecules in ligand-GR complexes (96). All of these works indicated that water molecules may be requisite for the binding of ligands to GR. Based on the above analyses, it is concluded that the changes in the ligand-nucleotide interactions induced by multiple mutations enhanced the binding ability of 6AP and HPA to GRs, implying that the aptamer domain activity of GR is extremely plastic and thus readily tunable by nucleotide mutations so as to meet cellular needs, which basically agrees with the experimental study of Stoddard *et al.* (25).

### Influence of mutations on dynamics of GR

**Figure 6.**
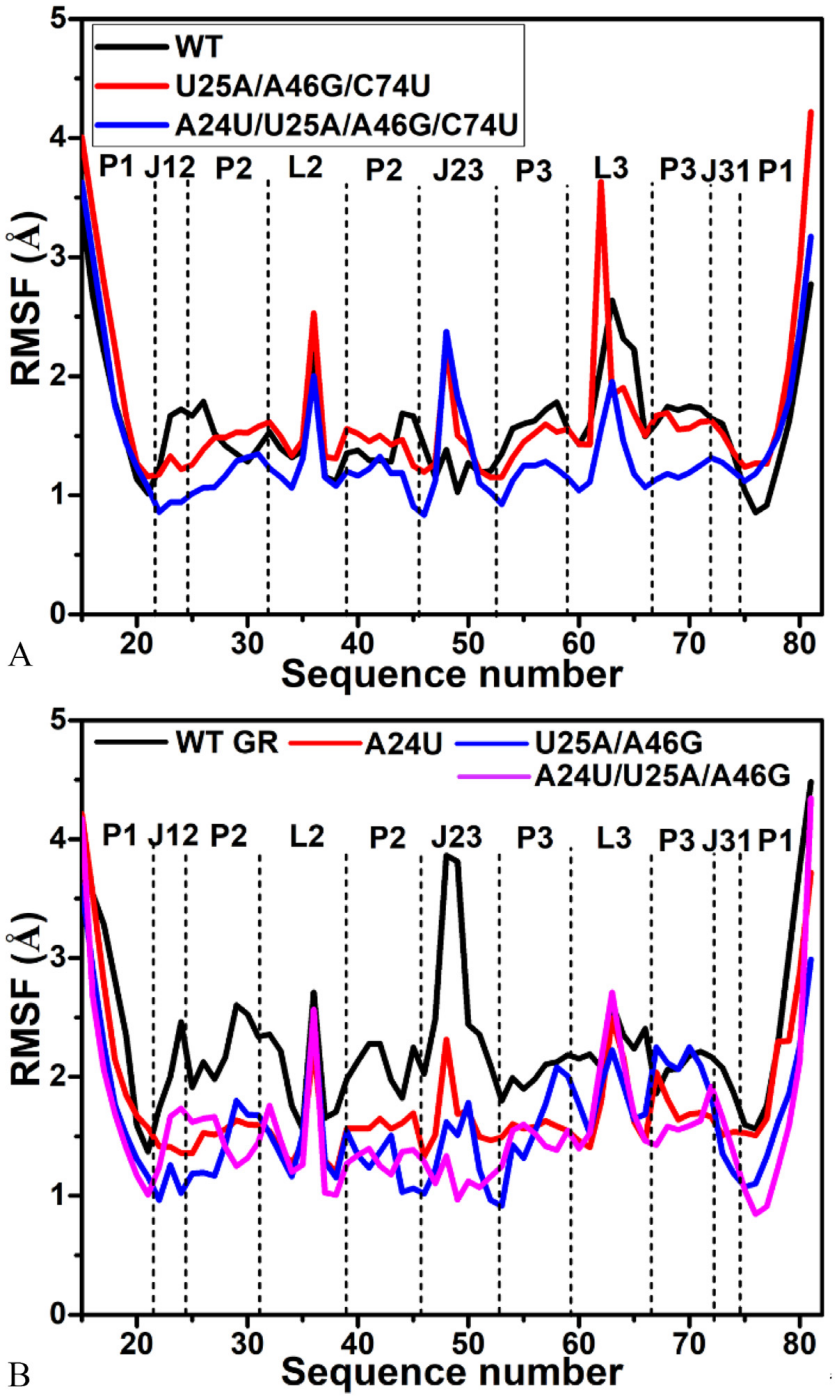
Root-mean-square fluctuation (RMSF) of atoms C′ in nucleotides of GR VS sequence number: (**A**) the WT and mutated GRs complexed with 6AP and (**B**) the WT and mutated GRs complexed with HPA.

To evaluate the influence of mutations on the flexibility of GRs, root-mean-square fluctuations (RMSFs) were computed by using C1′ atoms of nucleotides in GR based on the equilibrated MD trajectories (Figure 6). In the 6AP-GR complexes, the flexibility of nucleotides in GR is heavily affected by mutations (Figure 6A). Mutations U25A/A46G/C74U and A24U/U25A/A46G/C74U extremely strengthen the flexibility of J23 in GR and slightly enhance the flexibility of P1 in GRs. Mutations U25A/A46G/C74U and A24U/U25A/A46G/C74U generate opposite impact on P2 and L3 of GR. The former one strengthens the fluctuations of P2 and L3, while the latter one highly weakens the flexibility of P2 and L3 in GR. As a whole, the flexibility of the rest in GR is also restrained by mutations U25A/A46G/C74U and A24U/U25A/A46G/C74U, including J12 and J31. Interestingly, J12, J23 and J31 consist of binding pocket of 6AP to GR, thus the changes in the flexibility of J12, J23 and J31 caused by mutations certainly affect the binding ability of 6AP to GR. Similar to the 6AP-GR complexes, mutations A24U, U25A/A46G and A24U/U25A/A46G also give rise to the important effect on the flexibility of GR complexed with HPA (Figure 6B). Apart from L2 and L3, the flexibility of the rest part of GR is extremely constrained by mutations, especially for J12, J23 and J31 around the binding site of GR, which may regulate the binding ability of HPA to GR. The above analyses suggest that modest sequence alterations in nucleotides have a dramatic impact on flexibility of GR.

**Figure 7.**
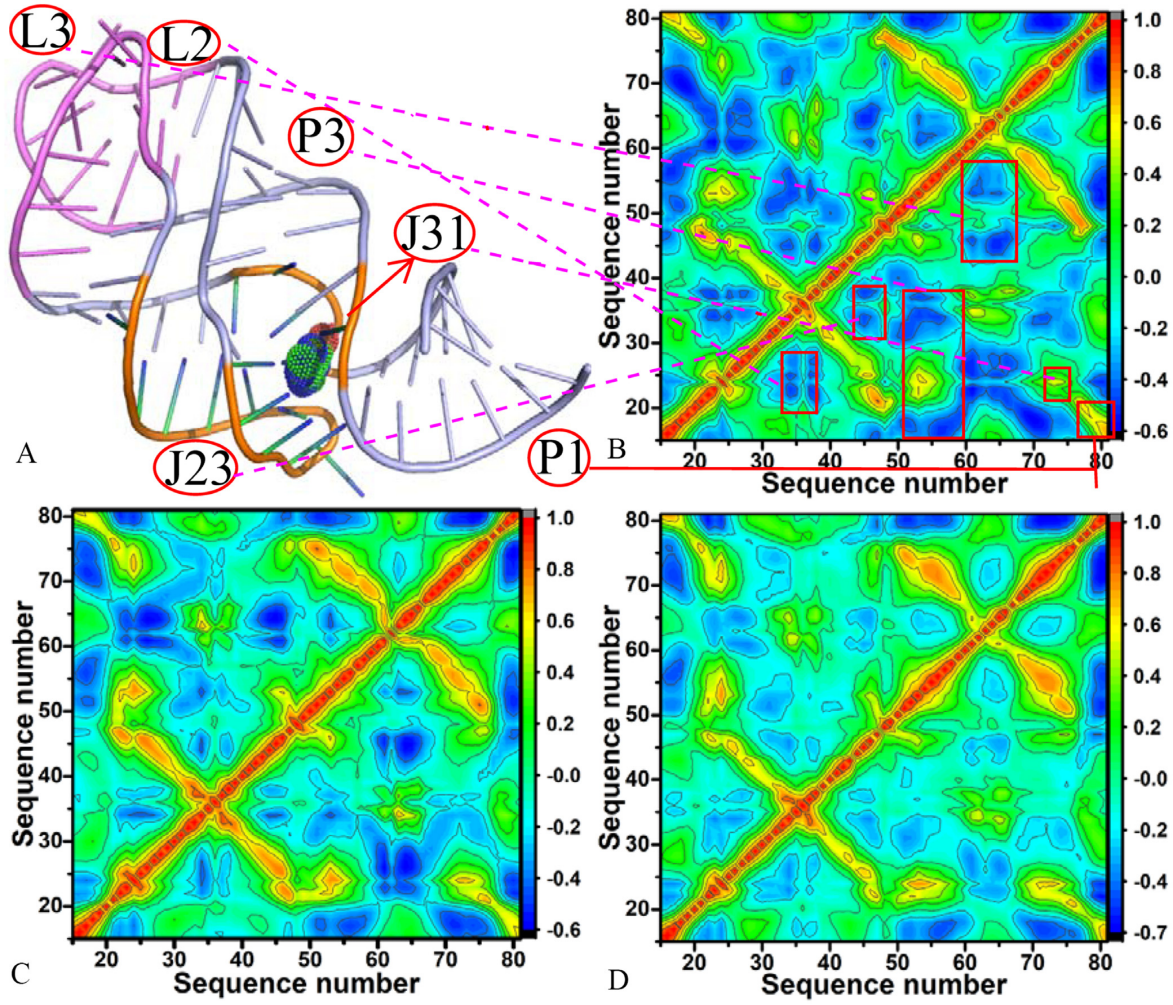
Relation between structure and correlation motions of GRs complexed with 6AP: (**A**) structure of GR; (**B**) correlation map of the WT GR; (**C**) correlation map of the mutated GR with U25A/A46G/C74U and (**D**) correlation map of the mutated GR with A24U/U25A/A46G/C74U

**Figure 8.**
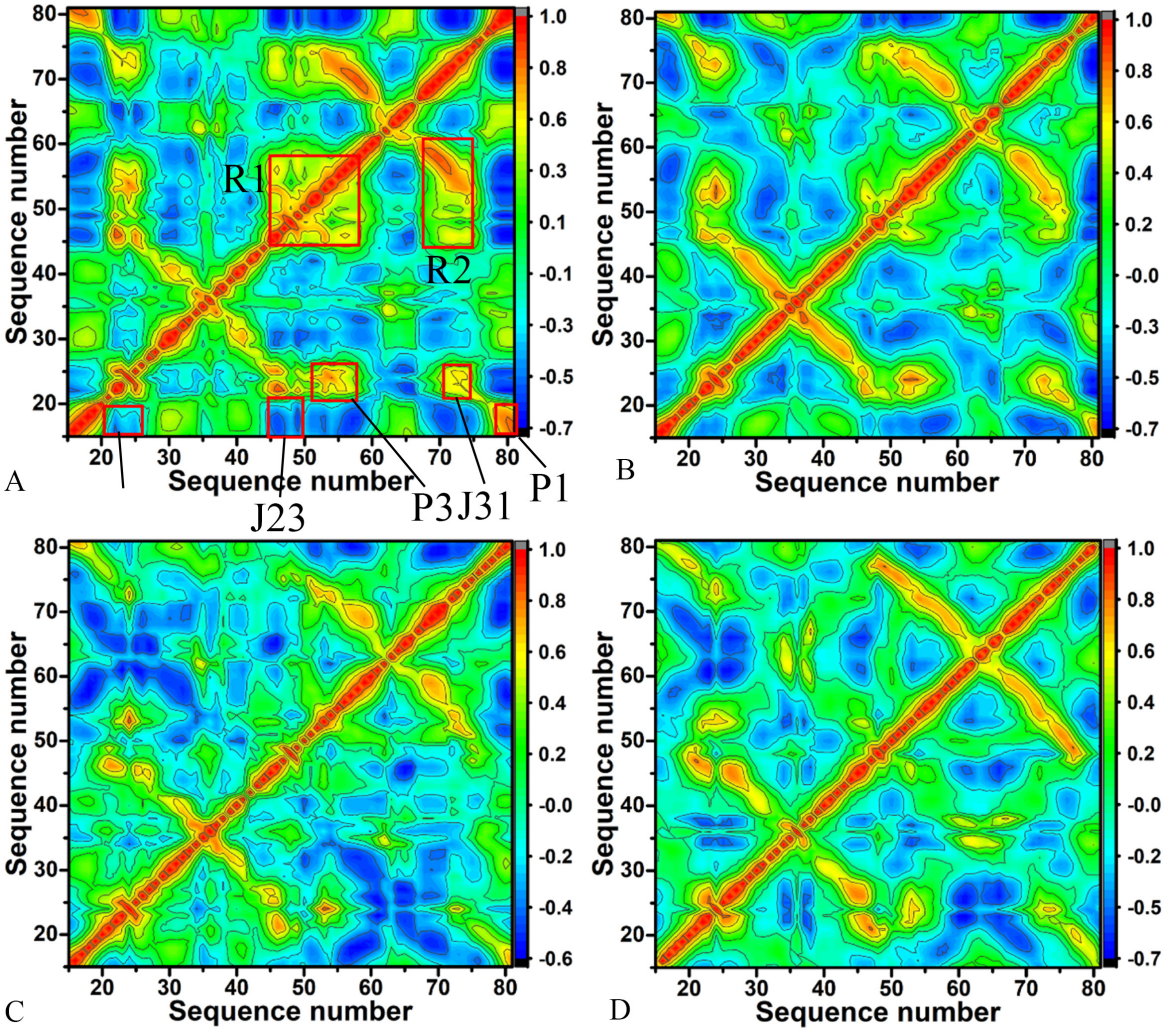
Cross-correlation maps between atoms C′ of nucleotides in GRs complexed with ligand HPA calculated by using the equilibrated MD trajectories: (**A**) the WT GR, (**B**) the mutated GR with A24U, (**C**) the mutated GR with U25A/A46G and (**D**) the mutated GR with A24U/U25A/A46G.

To obtain the details involving the influence of mutations on motion modes of GR, the correlation maps of C1′ atoms in nucleotides of GR were computed using the program CPPTRAJ in Amber and the results were depicted in Figure 7 and 8 in color-coded modes. The red and yellow reflect strongly correlated motions between specific nucleotides of GR, while the blue and dark blue are indicative of strongly anti-correlated motions between nucleotides. The diagonal regions represent the motion of a nucleotide to itself. It is found that mutations induce obvious changes on the motion modes of GR.

For the 6AP-WT GR complexes, the correlated motions mainly occur in the diagonal region (red and yellow), while the anti-correlated motion are mostly observed in the off-diagonal regions (Figure 7B). The structural components L2, J23, P3 and L3 of the WT GR respectively produce strongly anti-correlated motions relative to nucleotides 20–28, 30–37, 15–38 and 42–56, while P1 and J31 separately generate strongly correlated motions relative to nucleotides 15–20 and 20–25 (Figure 7A and B). Compared to the WT GR, mutations U25A/A46G/C74U not only weaken the anti-correlated motions of L2, J23, P3 and L3 separately relative to nucleotides 20–28, 30–37, 15–38 and 42–56, but also strengthen the correlated motions of P1 and J31 relative to nucleotides 15–20 and 20–25 (Figure 7C). Besides, mutations U25A/A46G/C74U also slightly strengthen the correlated movements between P3 and nucleotides 15–38. By comparison with the WT GR, it is found that mutations A24U/U25A/A46G/C74U extremely decrease the anti-correlated motions of L2, J23, P3 and L3 relative to nucleotides 20–28, 30–37, 15–38 and 42–56, moreover the anti-correlated motions in some certain regions of GR even disappear due to mutations A24U/U25A/A46G/C74U (Figure 7D). Meanwhile, the correlated movements of P3 and J31 relative to nucleotides 20–28 and 21–25 are heavily decreased.

For the HPA-WT GR complex, the structural components J12 and J23 of the WT GR produce strongly anti-correlated motions relative to nucleotides 15–20, while the components P1, P3 and J31 separately form strongly correlated motions relative to nucleotides 20–25, 20–25 and 15–20 (Figure 8A). It is also observed that strongly correlated movements occur in the regions R1 and R2, and the region R1 involves the structural components J23 and P3, while the region R2 mainly includes the structural component J31. The region R1 reflects the correlated motions of J23 and P3 relative to themselves and the region R2 mostly describes the correlated movements between J31 and nucleotides 20–25 (Figure 8A). Compared to the WT GR with HPA, mutation A24U not only heavily weakens the correlated motions of the regions R1 and R2, but also the anti-correlated motions of J12 and J23 relative to nucleotides 15–20 (Figure 8B). Although mutation A24U strengthens the correlated motions between J31 and nucleotides 20–25, A24U also weakens the correlated motions between P1 and nucleotides 20–25 (Figure 8B). AS shown in Figure 8C, mutations U25A/A46G produce obvious effect on motion modes of GR with HPA. Mutations U25A/A46G not only highly weaken the correlated motions of the regions R1 and R2, but also the correlated motions of P3, J31 and P1 separately relative to nucleotides 20–25, 20–25 and 15–20 compared to the WT GR. In addition, mutations U25A/A46G also obviously weaken the anti-correlated motions of J12 and J23 relative to nucleotides 15–20 (Figure 8C). According to Figure 8D, the correlated motions in the regions R1 and R2 and that of P3, J31 and P1 separately relative to nucleotides 20–25, 20–25 and 15–20 are extremely decreased by mutations A24U/U25A/A46G compared to the WT GR. Meanwhile, the anti-correlated movements of J12 and J23 relative to nucleotides 15–20 are also heavily reduced due to mutations A24U/U25A/A46G relative to the WT GR.

Based on the above analyses of RMSF, several mutations involved in this study produce obvious effect on the flexibility of GR, especially for J12, J23 and J31 around the binding site of ligands. The calculated correlation maps also suggest that mutations highly affect motion modes of GR and change the correlated extent of motions between nucleotides. These results show that modest sequence alterations in nucleotides can exert a dramatic influence on flexibility and dynamic behavior of GR, implying the aptamer domain activity of GR and binding ability can be easily regulated by nucleotide mutations, which basically agrees with the study of Stoddard *et al.* (25).

## DISCUSSIONS

In this work, 1-μs molecular dynamics simulations were performed on seven systems involving the WT and mutated GRs complexed with two ligands 6AP and HPA to probe the effect of mutations on dynamics behavior of GRs. In a summary, RMSD of atoms P, O3′, O5′ C3′, C4′ and C5′ in GRs calculated using the equilibrated MD trajectories indicate that all simulated systems reach the equilibrium after 200 ns of MD simulations. The averaged RMSD of seven systems are lower than 4.21 Å and the fluctuation range of RMSDs is also <1.13 Å, suggesting that the equilibrated MD trajectories can be reliably used to predict the binding affinities, selectivity of two ligands toward the WT and mutated GRs as well as dynamics changes of GRs due to mutations.

The cross-correlation analyses based on the equilibrated MD trajectories show that mutations change motion modes and internal dynamics of GR. Meanwhile, the calculated RMSFs of C1′ atoms in nucleotides of GR indicate that mutations highly affect the structural flexibility of GR. To better discuss the impact of mutations on conformations of GRs, cluster analysis was conducted on the last 600 ns of MD trajectories by using the iMWK-Means method, depicted in Supplementary Figures S5 and S6. The results show that mutations induce changes in cluster redistributions (including C1, C2 and C3) of GRs. The superimpositions of structures extracted from main clusters indicate that 6AP and HPA produce obvious slide and rotation in the binding pocket of the mutated GRs relative to the WT GR, which may affect the binding activity of GR. Meanwhile, PC analysis was also run on the equilibrated MD trajectories. Supplementary Figure S7 displays the function of eigenvalues as index of eigenvectors. In the complexes of the mutated GRs with HPA, mutations A24U, U25A/A46G and A24U/U25A/A46G weaken the motion strength of GR compared to the WT GR, while for that of the mutated GRs with 6AP, mutations U25A/A46G/C74U and A24U/U25A/A46G/C74U strengthen the motion of GR relative to the WT GR. The porcupine plots captured by the first eigenvector arising from PC analysis (Supplementary Figures S8 and S9) suggest that the mutations in this work exert significant influence on dynamics behavior of the regions P1, P2, J23 and J31 in GR. Free energy landscapes were built by using projections of MD trajectories on the eigenvectors of the first two principal components PC1 and PC2 and structures corresponding to different free energy basins E1 and E2 were superimposed to understand conformations of GR due mutations (Supplementary Figures S10 and S11). The results indicate that mutations not only induce the redistribution of free energy basins, but also lead to obvious slide and rotation of two ligands in the binding pocket of the mutated GRs relative to the WT GR, which may tune binding ability of ligands to GR. Therefore, the results of cluster and PC analyses further support the conclusion that the aptamer domain activity of GR is extremely plastic and thus readily tunable by nucleotide mutations.

FEP and MM-GBSA methods were applied to compute binding free energies of 6AP and HPA to the WT and mutated GRs so as to comparatively evaluate influence of mutations on binding ability of ligands to GR. The results not only suggest that binding free energies predicted by two different methods exist encouraging correlations, but also clarify that mutations in the current works strengthen bindings of ligands to GR. Meanwhile, mutations generate important influence on enthalpy changes and entropy changes. As shown in Table 2, the enthalpy change ($\Delta H$) and entropy change ($ - {{T}}\Delta S$) of HPA-GR binding is respectively increased by 0.91 kcal/mol and decreased by 0.15 kcal/mol due to A24U compared to the WT GR, totally leading to stronger association of HPA with the mutated GR. By comparison with the WT GR, despite almost no change in binding enthalpy of HPA to GR caused by U25A/A46G, a decrease of 1.69 kcal/mol in binding entropy of HPA to GR induced by U25A/A46G provides main contribution for stronger bindings of HPA to the U25A/A46G mutated GR. Although the entropy change of HPA-GR binding is increased by 0.8 kcal/mol due to A24U/U25A/A46G relative to the WT GR, this unfavorable change is completely compensated by an increase of 6.24 kcal/mol in enthalpy change. By comparison with the 6AP-WT GR, mutations U25A/A46G/C74U and A24U/U25A/A46G/C74U not only increase the enthalpy of 6AP-GR binding, but also decrease the entropy change, thus two mutations highly strengthen binding of 6AP to the mutated GRs. It is observed from Table 2 that binding strength of 6AP to the WT GR is much stronger than HPA, indicating a more favorable binding selectivity of 6AP toward the WT GR over HPA. The comparison of separate free energy components computed by MM-GBSA method shows that the difference in van der Waals interactions and nonpolar solvation energy is small, while the unfavorable polar interaction of 6AP with the WT GR is reduced by 10.38 kcal/mol compared to that of HPA with the WT GR, which is mostly responsible for driving contributions to more favorable selectivity of 6AP toward the WT GR than HPA.

The ligand-nucleotide interaction spectrum calculated by using residue-based free energy decomposition method demonstrate that mutations highly affect interactions of ligands with key nucleotides U22, U51 and C74 in GR, which provides significant contributions for the changes of binding ability of ligands induced by mutations. More importantly, the mutations involved in the current study induce the formation of new additional hydrogen bonding interactions of HPA and 6AP with GR, moreover multiple published works also observed this phenomenon (19,22,25). These changes of ligand-nucleotide interactions should be responsible for certain contributions to conformational changes of GRs due to mutations and play significant role in the plasticity and tuning of the aptamer domain activity of GR. We expect that this study contribute molecular basis and dynamics information for understanding function of GR and possibility as potential drug targets for antibacterial.

## Supplementary Material

gkz499_Supplemental_File
