# Supplementary material for: Effect of mutations on binding of ligands to guanine riboswitch probed by free energy perturbation and molecular dynamics simulations
Source: Nucleic Acids Res. 2019 Jun 7;47(13):6618–31. doi: 10.1093/nar/gkz499 (PMC6649850; doi:10.1093/nar/gkz499)
Supplement: gkz499_Supplemental_File [file gkz499_supplemental_file.pdf]

Effect of mutations on binding of ligands to guanine riboswitch probed by free energy perturbation and molecular dynamics simulations

Jianzhong Chen[1,*], Xingyu Wang[2], Laixue Pang[1], John Z.H. Zhang[2,3], Tong Zhu[2,3,*]

[1]School of Science, Shandong Jiaotong University, Jinan 250357 China, [2]NYU-ECNU Center for Computational Chemistry at NYU Shanghai, Shanghai, 200062, China, [3]Shanghai Engineering Research Center of Molecular Therapeutics & New Drug Development, School of Chemistry and Molecular Engineering, East China Normal University, Shanghai, 200062, China

ORCID:

Jianzhong Chen: 0000-0003-1558-4398

John Z. H. Zhang: 0000-0003-4612-1863

Tong Zhu: 0000-0001-7472-3736

[*] To whom correspondence should be addressed. E-mail: chenjianzhong1970@163.com and jzchen@sdjtu.edu.cn. Correspondence may also be addressed to Tong Zhu. Tel: E-mail: tzhu@lps.ecun.edu.cn.

File S1. Force field parameters of HPA for Amber

```
%VERSION  VERSION_STAMP = V0001.000  DATE = 06/08/18  09:24:49
%FLAG TITLE
%FORMAT(20a4)
lig
%FLAG POINTERS
%FORMAT(10I8)
      14       7       4      11       8      15      17      23       0       0
      64       1      11      15      23      11      19       9       9       0
       0       0       0       0       0       0       0       0      14       0
       0
%FLAG ATOM_NAME
%FORMAT(20a4)
N1  C2  N3  C4  C5  C6  O6  N7  C8  N9  H1  H2  H3  H4
%FLAG CHARGE
%FORMAT(5E16.8)
 -8.84510442E+00  8.56083654E+00 -1.14563600E+01  1.00823986E+01 -5.98055886E+00
  1.33587681E+01 -1.15438270E+01 -5.04575487E+00  7.93398942E+00 -1.15347159E+01
  6.22291545E+00  1.07147124E+00  5.89855851E+00  1.27738323E+00
%FLAG ATOMIC_NUMBER
%FORMAT(10I8)
       7       6       7       6       6       6       8       7       6       7
       1       1       1       1
%FLAG MASS
%FORMAT(5E16.8)
  1.40100000E+01  1.20100000E+01  1.40100000E+01  1.20100000E+01  1.20100000E+01
  1.20100000E+01  1.60000000E+01  1.40100000E+01  1.20100000E+01  1.40100000E+01
  1.00800000E+00  1.00800000E+00  1.00800000E+00  1.00800000E+00
%FLAG ATOM_TYPE_INDEX
%FORMAT(10I8)
       1       2       3       2       2       2       4       5       2       3
       6       7       6       7
%FLAG NUMBER_EXCLUDED_ATOMS
%FORMAT(10I8)
       9       8       8       9       8       7       2       4       3       2
       1       1       1       1
%FLAG NONBONDED_PARM_INDEX
%FORMAT(10I8)
       1       2       4       7      11      16      22       2       3       5
```

```
       8      12      17      23       4       5       6       9      13      18
      24       7       8       9      10      14      19      25      11      12
      13      14      15      20      26      16      17      18      19      20
      21      27      22      23      24      25      26      27      28
%FLAG RESIDUE_LABEL
%FORMAT(20a4)
HPA
%FLAG RESIDUE_POINTER
%FORMAT(10I8)
       1
%FLAG BOND_FORCE_CONSTANT
%FORMAT(5E16.8)
  3.53830000E+02  3.56210000E+02  5.27310000E+02  4.50710000E+02  4.03490000E+02
  3.69100000E+02  4.16130000E+02  3.54490000E+02  2.95350000E+02  6.52570000E+02
  5.35140000E+02
%FLAG BOND_EQUIL_VALUE
%FORMAT(5E16.8)
  1.38100000E+00  1.37900000E+00  1.01300000E+00  1.31700000E+00  1.08200000E+00
  1.36900000E+00  1.37300000E+00  1.38000000E+00  1.46800000E+00  1.21800000E+00
  1.01000000E+00
%FLAG ANGLE_FORCE_CONSTANT
%FORMAT(5E16.8)
  1.10304000E+02  6.27370000E+01  8.71450000E+01  1.13811000E+02  6.65790000E+01
  4.82850000E+01  7.38710000E+01  6.24610000E+01  9.10570000E+01  1.07603000E+02
  9.26530000E+01  6.71870000E+01  7.04920000E+01  4.71010000E+01  8.67360000E+01
  4.86910000E+01  6.86950000E+01  1.15504000E+02  6.12260000E+01
%FLAG ANGLE_EQUIL_VALUE
%FORMAT(5E16.8)
  2.14675590E+00  2.01934681E+00  1.96698691E+00  2.14762857E+00  2.15146829E+00
  2.08148056E+00  1.84114862E+00  2.19073822E+00  1.94866095E+00  2.19387981E+00
  1.86732857E+00  2.11795796E+00  1.91811767E+00  2.19038915E+00  2.16298747E+00
  2.05163542E+00  1.98679641E+00  1.95860933E+00  2.12144862E+00
%FLAG DIHEDRAL_FORCE_CONSTANT
%FORMAT(5E16.8)
  4.75000000E+00  2.87500000E+00  2.50000000E+00  1.65000000E+00  4.00000000E+00
  1.70000000E+00  2.00000000E+00  1.10000000E+00  1.05000000E+01
%FLAG DIHEDRAL_PERIODICITY
%FORMAT(5E16.8)
  2.00000000E+00  2.00000000E+00  2.00000000E+00  2.00000000E+00  2.00000000E+00
  2.00000000E+00  1.00000000E+00  2.00000000E+00  2.00000000E+00
```

```
%FLAG DIHEDRAL_PHASE
%FORMAT(5E16.8)
  3.14159400E+00  3.14159400E+00  3.14159400E+00  3.14159400E+00  3.14159400E+00
  3.14159400E+00  0.00000000E+00  3.14159400E+00  3.14159400E+00
%FLAG SCEE_SCALE_FACTOR
%FORMAT(5E16.8)
  1.20000000E+00  1.20000000E+00  1.20000000E+00  1.20000000E+00  1.20000000E+00
  1.20000000E+00  1.20000000E+00  0.00000000E+00  0.00000000E+00
%FLAG SCNB_SCALE_FACTOR
%FORMAT(5E16.8)
  2.00000000E+00  2.00000000E+00  2.00000000E+00  2.00000000E+00  2.00000000E+00
  2.00000000E+00  2.00000000E+00  0.00000000E+00  0.00000000E+00
%FLAG SOLTY
%FORMAT(5E16.8)
  0.00000000E+00  0.00000000E+00  0.00000000E+00  0.00000000E+00  0.00000000E+00
  0.00000000E+00  0.00000000E+00  0.00000000E+00  0.00000000E+00
%FLAG LENNARD_JONES_ACOEF
%FORMAT(5E16.8)
  7.02078680E+05  7.01106848E+05  6.96551276E+05  7.76656876E+05  7.69642101E+05
  8.49322032E+05  5.15491878E+05  5.17507491E+05  5.74782316E+05  3.76435470E+05
  8.22085792E+05  8.20152428E+05  9.08092643E+05  6.04208667E+05  9.62429652E+05
  1.52360475E+03  1.71458823E+03  2.01475241E+03  9.87857350E+02  1.82491629E+03
  1.34728399E-01  5.06296765E+04  5.22195041E+04  5.87815925E+04  3.59538577E+04
  5.96470418E+04  5.02830284E+01  2.97255425E+03
%FLAG LENNARD_JONES_BCOEF
%FORMAT(5E16.8)
  6.77820248E+02  5.97113873E+02  5.24668528E+02  6.20851625E+02  5.44830041E+02
  5.65406768E+02  5.64804067E+02  4.98871431E+02  5.19385932E+02  4.69350655E+02
  7.75261695E+02  6.82622272E+02  7.09588248E+02  6.46321327E+02  8.86629877E+02
  1.57004457E+01  1.46824710E+01  1.57231024E+01  1.22938581E+01  1.81620725E+01
  7.34107347E-02  1.01949445E+02  9.12729468E+01  9.56653235E+01  8.35449674E+01
  1.16962018E+02  1.59752321E+00  1.38359132E+01
%FLAG BONDS_INC_HYDROGEN
%FORMAT(10I8)
       0      30       3       3      33       5      21      36      11      24
      39       5
%FLAG BONDS_WITHOUT_HYDROGEN
%FORMAT(10I8)
       0       3       1       0      15       2       3       6       4       6
       9       6       9      12       7       9      27       6      12      21
```

```
       8      12      15       9      15      18      10      21      24       8
      24      27       4
%FLAG ANGLES_INC_HYDROGEN
%FORMAT(10I8)
       0       3      33       2       3       0      30       6       6       3
      33       8      12      21      36      14      15       0      30      16
      21      24      39      19      24      21      36      14      27      24
      39       8
%FLAG ANGLES_WITHOUT_HYDROGEN
%FORMAT(10I8)
       0       3       6       1       0      15      12       3       0      15
      18       4       3       0      15       5       3       6       9       7
       6       9      12       9       6       9      27      10       9      12
      21      11       9      12      15      12       9      27      24       7
      12       9      27       9      12      21      24      13      12      15
      18      15      15      12      21      17      21      24      27      18
%FLAG DIHEDRALS_INC_HYDROGEN
%FORMAT(10I8)
      30       0       3       6       4       9       6       3      33       1
       9      12      21      36       6       9      27      24      39       1
      12      21      24      39       6      30       0      15      12       3
      15       0       3      33       4      15      12      21      36       6
      30       0      15      18       7      30       0     -15      18       3
      27      24      21      36       6      30       0       3      33       4
      36      21      24      39       6      30       0      -3     -15       8
      33       0      -3      -6       8      12      24     -21     -36       8
      39      21     -24     -27       8
%FLAG DIHEDRALS_WITHOUT_HYDROGEN
%FORMAT(10I8)
       0       3       6       9       1       0      15     -12       9       2
       0      15      12      21       2       3       0      15      12       3
       3       0      15      18       3       3       6      -9      12       1
       3       6       9      27       1      15       0       3       6       4
       6       9      12      21       5       6       9     -12      15       5
       6       9      27      24       1       9      12     -21      24       6
       9      12      15      18       2       9      27     -24      21       1
      12       9     -27      24       1      12      21     -24      27       6
      15      12       9      27       5      15      12      21      24       6
      18      15      12      21       2      21      12      -9      27       5
      12       6      -9     -27       8      15       9     -12     -21       8
```

```
      12       0     -15     -18       9
%FLAG EXCLUDED_ATOMS_LIST
%FORMAT(10I8)
       2       3       4       5       6       7       8      11      12       3
       4       5       6       7      10      11      12       4       5       6
       8       9      10      11      12       5       6       7       8       9
      10      12      13      14       6       7       8       9      10      11
      13      14       7       8       9      10      11      12      13       8
      11       9      10      13      14      10      13      14      13      14
      12       0      14       0
%FLAG HBOND_ACOEF
%FORMAT(5E16.8)

%FLAG HBOND_BCOEF
%FORMAT(5E16.8)

%FLAG HBCUT
%FORMAT(5E16.8)

%FLAG AMBER_ATOM_TYPE
%FORMAT(20a4)
n   cc  nd  cd  cc  c   o   na  cc  nd  hn  h5  hn  h5
%FLAG TREE_CHAIN_CLASSIFICATION
%FORMAT(20a4)
BLA BLA BLA BLA BLA BLA BLA BLA BLA BLA BLA BLA BLA BLA
%FLAG JOIN_ARRAY
%FORMAT(10I8)
       0       0       0       0       0       0       0       0       0       0
       0       0       0       0
%FLAG IROTAT
%FORMAT(10I8)
       0       0       0       0       0       0       0       0       0       0
       0       0       0       0
%FLAG RADIUS_SET
%FORMAT(1a80)
modified Bondi radii (mbondi)
%FLAG RADII
%FORMAT(5E16.8)
  1.55000000E+00  1.70000000E+00  1.55000000E+00  1.70000000E+00  1.70000000E+00
  1.70000000E+00  1.50000000E+00  1.55000000E+00  1.70000000E+00  1.55000000E+00
```

```
   1.30000000E+00  1.30000000E+00  1.30000000E+00  1.30000000E+00
%FLAG SCREEN
%FORMAT(5E16.8)
   7.90000000E-01  7.20000000E-01  7.90000000E-01  7.20000000E-01  7.20000000E-01
   7.20000000E-01  8.50000000E-01  7.90000000E-01  7.20000000E-01  7.90000000E-01
   8.50000000E-01  8.50000000E-01  8.50000000E-01  8.50000000E-01
%FLAG IPOL
%FORMAT(1I8)
       0
```

File S2. Force field parameters of 6AP for Amber

```
%VERSION  VERSION_STAMP = V0001.000  DATE = 06/13/18  10:13:21
%FLAG TITLE
%FORMAT(20a4)
6AP
%FLAG POINTERS
%FORMAT(10I8)
      17       6       6      12      10      17      18      26       0       0
      76       1      12      17      26      10      18       7       8       0
       0       0       0       0       0       0       0       0      17       0
       0
%FLAG ATOM_NAME
%FORMAT(20a4)
N1  C2  N2  N3  C4  C5  C6  N6  N7  C8  N9  H1  H2  H3  H4  H5  H6
%FLAG CHARGE
%FORMAT(5E16.8)
  -1.53613989E+01  1.67626938E+01 -1.68829610E+01 -1.44867285E+01  8.97994944E+00
  -4.92366546E+00  1.43810392E+01 -1.74296300E+01 -9.71430813E+00  7.06478571E+00
  -7.07025240E+00  7.74083304E+00  7.74083304E+00  8.08705674E+00  8.08705674E+00
   1.29013884E+00  5.75278011E+00
%FLAG ATOMIC_NUMBER
%FORMAT(10I8)
       7       6       7       7       6       6       6       7       7       6
       7       1       1       1       1       1       1
%FLAG MASS
%FORMAT(5E16.8)
   1.40100000E+01  1.20100000E+01  1.40100000E+01  1.40100000E+01  1.20100000E+01
   1.20100000E+01  1.20100000E+01  1.40100000E+01  1.40100000E+01  1.20100000E+01
```

```
  1.40100000E+01  1.00800000E+00  1.00800000E+00  1.00800000E+00  1.00800000E+00
  1.00800000E+00  1.00800000E+00
%FLAG ATOM_TYPE_INDEX
%FORMAT(10I8)
       1       2       3       1       2       2       2       3       1       2
       4       5       5       5       5       6       5
%FLAG NUMBER_EXCLUDED_ATOMS
%FORMAT(10I8)
      12       9       5       9       8       9       6       3       4       3
       2       1       1       1       1       1       1
%FLAG NONBONDED_PARM_INDEX
%FORMAT(10I8)
       1       2       4       7      11      16       2       3       5       8
      12      17       4       5       6       9      13      18       7       8
       9      10      14      19      11      12      13      14      15      20
      16      17      18      19      20      21
%FLAG RESIDUE_LABEL
%FORMAT(20a4)
6AP
%FLAG RESIDUE_POINTER
%FORMAT(10I8)
       1
%FLAG BOND_FORCE_CONSTANT
%FORMAT(5E16.8)
  4.14240000E+02  3.47060000E+02  5.29460000E+02  3.78570000E+02  3.49520000E+02
  3.94620000E+02  4.50710000E+02  3.54490000E+02  4.03680000E+02  5.35140000E+02
%FLAG BOND_EQUIL_VALUE
%FORMAT(5E16.8)
  1.33900000E+00  1.38600000E+00  1.01200000E+00  1.39800000E+00  1.38400000E+00
  1.35200000E+00  1.31700000E+00  1.38000000E+00  1.08200000E+00  1.01000000E+00
%FLAG ANGLE_FORCE_CONSTANT
%FORMAT(5E16.8)
  1.12063000E+02  1.09369000E+02  8.68490000E+01  7.03560000E+01  4.87870000E+01
  1.07575000E+02  6.87670000E+01  8.76350000E+01  6.93770000E+01  4.69790000E+01
  8.71670000E+01  8.61630000E+01  7.46080000E+01  1.15504000E+02  6.24620000E+01
  4.71010000E+01  6.12270000E+01  3.95190000E+01
%FLAG ANGLE_EQUIL_VALUE
%FORMAT(5E16.8)
  2.04098890E+00  2.22110696E+00  2.14570870E+00  2.04587583E+00  2.02580453E+00
  2.21813990E+00  2.09474507E+00  2.08950908E+00  1.97484090E+00  2.19108728E+00
```

```
  2.06542352E+00  2.11097664E+00  1.83050210E+00  1.95860933E+00  2.19073822E+00
  2.19038915E+00  2.12144862E+00  2.00922390E+00
%FLAG DIHEDRAL_FORCE_CONSTANT
%FORMAT(5E16.8)
  1.05000000E+00  4.80000000E+00  3.62500000E+00  3.00000000E-01  1.70000000E+00
  4.75000000E+00  1.10000000E+00
%FLAG DIHEDRAL_PERIODICITY
%FORMAT(5E16.8)
  2.00000000E+00  2.00000000E+00  2.00000000E+00  2.00000000E+00  2.00000000E+00
  2.00000000E+00  2.00000000E+00
%FLAG DIHEDRAL_PHASE
%FORMAT(5E16.8)
  3.14159400E+00  3.14159400E+00  3.14159400E+00  3.14159400E+00  3.14159400E+00
  3.14159400E+00  3.14159400E+00
%FLAG SCEE_SCALE_FACTOR
%FORMAT(5E16.8)
  1.20000000E+00  1.20000000E+00  1.20000000E+00  1.20000000E+00  1.20000000E+00
  1.20000000E+00  0.00000000E+00
%FLAG SCNB_SCALE_FACTOR
%FORMAT(5E16.8)
  2.00000000E+00  2.00000000E+00  2.00000000E+00  2.00000000E+00  2.00000000E+00
  2.00000000E+00  0.00000000E+00
%FLAG SOLTY
%FORMAT(5E16.8)
  0.00000000E+00  0.00000000E+00  0.00000000E+00  0.00000000E+00  0.00000000E+00
  0.00000000E+00  0.00000000E+00  0.00000000E+00
%FLAG LENNARD_JONES_ACOEF
%FORMAT(5E16.8)
  8.49322032E+05  7.69642101E+05  6.96551276E+05  9.05243612E+05  8.17329084E+05
  9.54790629E+05  9.08092643E+05  8.20152428E+05  9.58637891E+05  9.62429652E+05
  2.01475241E+03  1.71458823E+03  1.79157166E+03  1.82491629E+03  1.34728399E-01
  5.87815925E+04  5.22195041E+04  5.91752946E+04  5.96470418E+04  5.02830284E+01
  2.97255425E+03
%FLAG LENNARD_JONES_BCOEF
%FORMAT(5E16.8)
  5.65406768E+02  5.44830041E+02  5.24668528E+02  7.17661669E+02  6.90283231E+02
  9.06156687E+02  7.09588248E+02  6.82622272E+02  8.96356641E+02  8.86629877E+02
  1.57231024E+01  1.46824710E+01  1.82287422E+01  1.81620725E+01  7.34107347E-02
  9.56653235E+01  9.12729468E+01  1.18009316E+02  1.16962018E+02  1.59752321E+00
  1.38359132E+01
```

```
%FLAG BONDS_INC_HYDROGEN
%FORMAT(10I8)
       6      33       3       6      36       3      21      39       3      21
      42       3      27      45       9      30      48      10
%FLAG BONDS_WITHOUT_HYDROGEN
%FORMAT(10I8)
       0       3       1       0      18       1       3       6       2       3
       9       1       9      12       1      12      15       4      12      30
       5      15      18       4      15      24       6      18      21       2
      24      27       7      27      30       8
%FLAG ANGLES_INC_HYDROGEN
%FORMAT(10I8)
       3       6      33       5       3       6      36       5      12      30
      48      10      18      21      39       5      18      21      42       5
      24      27      45      15      27      30      48      16      30      27
      45      17      33       6      36      18      39      21      42      18
%FLAG ANGLES_WITHOUT_HYDROGEN
%FORMAT(10I8)
       0       3       6       1       0       3       9       2       0      18
      15       3       0      18      21       1       3       0      18       4
       3       9      12       4       6       3       9       1       9      12
      15       3       9      12      30       6      12      15      18       7
      12      15      24       8      12      30      27       9      15      12
      30      11      15      18      21      12      15      24      27      13
      18      15      24       8      24      27      30      14
%FLAG DIHEDRALS_INC_HYDROGEN
%FORMAT(10I8)
       0       3       6      33       1       0       3       6      36       1
       0      18      21      39       1       0      18      21      42       1
       9       3       6      33       1       9       3       6      36       1
       9      12      30      48       4      12      30      27      45       5
      15      12      30      48       4      15      18      21      39       1
      15      18      21      42       1      15      24      27      45       6
      24      27      30      48       5      45      27      30      48       5
       3      33      -6     -36       7      18      39     -21     -42       7
      45      30     -27     -24       7      12      27     -30     -48       7
%FLAG DIHEDRALS_WITHOUT_HYDROGEN
%FORMAT(10I8)
       0       3       9      12       2       0      18     -15      12       3
       0      18      15      24       3       3       0      18      15       2
```

```
       3       0      18      21       2       3       9     -12      15       2
       3       9      12      30       2      18       0       3       6       2
       6       3       9      12       2      18       0       3       9       2
       9      12     -15      18       3       9      12      15      24       3
       9      12      30      27       4      12      15      18      21       3
      12      15     -24      27       2      12      30     -27      24       5
      15      12     -30      27       4      15      24     -27      30       6
      18      15      12      30       3      18      15      24      27       2
      21      18      15      24       3      24      15     -12      30       3
       0       9      -3      -6       7      15      30     -12      -9       7
      12      18     -15     -24       7      15       0     -18     -21       7
%FLAG EXCLUDED_ATOMS_LIST
%FORMAT(10I8)
       2       3       4       5       6       7       8       9      12      13
      14      15       3       4       5       6       7       8      11      12
      13       4       5       7      12      13       5       6       7       9
      10      11      12      13      17       6       7       8       9      10
      11      16      17       7       8       9      10      11      14      15
      16      17       8       9      10      11      14      15       9      14
      15      10      11      16      17      11      16      17      16      17
      13       0      15       0      17       0
%FLAG HBOND_ACOEF
%FORMAT(5E16.8)

%FLAG HBOND_BCOEF
%FORMAT(5E16.8)

%FLAG HBCUT
%FORMAT(5E16.8)

%FLAG AMBER_ATOM_TYPE
%FORMAT(20a4)
nb  ca  nh  nb  ca  ca  ca  nh  nc  cd  na  hn  hn  hn  hn  h5  hn
%FLAG TREE_CHAIN_CLASSIFICATION
%FORMAT(20a4)
BLA BLA BLA BLA BLA BLA BLA BLA BLA BLA BLA BLA BLA BLA BLA BLA BLA
%FLAG JOIN_ARRAY
%FORMAT(10I8)
       0       0       0       0       0       0       0       0       0       0
       0       0       0       0       0       0       0
```

```
%FLAG IROTAT
%FORMAT(10I8)
       0       0       0       0       0       0       0       0       0       0
       0       0       0       0       0       0       0
%FLAG RADIUS_SET
%FORMAT(1a80)
modified Bondi radii (mbondi)
%FLAG RADII
%FORMAT(5E16.8)
  1.55000000E+00  1.70000000E+00  1.55000000E+00  1.55000000E+00  1.70000000E+00
  1.70000000E+00  1.70000000E+00  1.55000000E+00  1.55000000E+00  1.70000000E+00
  1.55000000E+00  1.30000000E+00  1.30000000E+00  1.30000000E+00  1.30000000E+00
  1.30000000E+00  1.30000000E+00
%FLAG SCREEN
%FORMAT(5E16.8)
  7.90000000E-01  7.20000000E-01  7.90000000E-01  7.90000000E-01  7.20000000E-01
  7.20000000E-01  7.20000000E-01  7.90000000E-01  7.90000000E-01  7.20000000E-01
  7.90000000E-01  8.50000000E-01  8.50000000E-01  8.50000000E-01  8.50000000E-01
  8.50000000E-01  8.50000000E-01
%FLAG IPOL
%FORMAT(1I8)
       0
```

File S3. Force field parameters of HPA for Gromacs:

```
; lig.top created by rdparm2gmx.pl 2018 年 02 月 05 日 星期一 19:51:02 CST

; Include forcefield parameters
#include "amber99sb-ildn.ff/forcefield.itp"

;[ defaults ]
; nbfunc        comb-rule       gen-pairs       fudgeLJ fudgeQQ
;1              2               yes             0.5     0.8333

[ atomtypes ]
;name   bond_type     mass     charge   ptype          sigma        epsilon
o              o     0.0000    0.0000   A     2.95992e-01    8.78640e-01
n              n     0.0000    0.0000   A     3.25000e-01    7.11280e-01
hn            hn     0.0000    0.0000   A     1.06908e-01    6.56888e-02
```

```
c              c       0.0000   0.0000   A    3.39967e-01   3.59824e-01
cd             cd      0.0000   0.0000   A    3.39967e-01   3.59824e-01
nd             nd      0.0000   0.0000   A    3.25000e-01   7.11280e-01
cc             cc      0.0000   0.0000   A    3.39967e-01   3.59824e-01
na             na      0.0000   0.0000   A    3.25000e-01   7.11280e-01
h5             h5      0.0000   0.0000   A    2.42146e-01   6.27600e-02

[ moleculetype ]
; Name            nrexcl
  HPA              3

[ atoms ]
;   nr       type  resnr residue  atom   cgnr     charge       mass  typeB    chargeB
     1          n      1    HPA     N1      1   -0.48540  14.000000
     2         cc      1    HPA     C2      2    0.46980  12.000000
     3         nd      1    HPA     N3      3   -0.62870  14.000000
     4         cd      1    HPA     C4      4    0.55330  12.000000
     5         cc      1    HPA     C5      5   -0.32820  12.000000
     6          c      1    HPA     C6      6    0.73310  12.000000
     7          o      1    HPA     O6      7   -0.63350  16.000000
     8         na      1    HPA     N7      8   -0.27690  14.000000
     9         cc      1    HPA     C8      9    0.43540  12.000000
    10         nd      1    HPA     N9     10   -0.63300  14.000000
    11         hn      1    HPA     H1     11    0.34150   1.000000
    12         h5      1    HPA     H2     12    0.05880   1.000000
    13         hn      1    HPA     H3     13    0.32370   1.000000
    14         h5      1    HPA     H4     14    0.07010   1.000000

[ bonds ]
;   ai     aj funct   r   k
     1     11     1   1.0090e-01   3.4326e+05
     2     12     1   1.0790e-01   2.9790e+05
     8     13     1   1.0110e-01   3.4024e+05
     9     14     1   1.0790e-01   2.9790e+05
     1      2     1   1.3800e-01   3.5648e+05
     1      6     1   1.3450e-01   4.0016e+05
     2      3     1   1.3350e-01   4.1388e+05
     3      4     1   1.3760e-01   3.6116e+05
     4     10     1   1.3760e-01   3.6116e+05
     4      5     1   1.3710e-01   4.2175e+05
```

```
    5      8      1   1.3710e-01   3.6719e+05
    5      6      1   1.4620e-01   3.1581e+05
    6      7      1   1.2140e-01   5.4225e+05
    8      9      1   1.3710e-01   3.6719e+05
    9     10      1   1.3350e-01   4.1388e+05

[ pairs ]
;   ai     aj funct
     11      3      1
      4     12      1
      4     14      1
      4     13      1
      5     14      1
     11      5      1
      6     12      1
      6     13      1
     11      7      1
     10     13      1
     11     12      1
     13     14      1
      1      4      1
      1      8      1
      2      5      1
      2      7      1
      2     10      1
      6      3      1
      3      9      1
      3      8      1
      4      7      1
      6     10      1
      6      9      1
      7      8      1

[ angles ]
;   ai     aj     ak funct   theta     cth
    1      2     12      1   1.1561e+02   4.2568e+02
    2      1     11      1   1.1871e+02   4.0233e+02
    3      2     12      1   1.2538e+02   4.1949e+02
    5      8     13      1   1.2566e+02   3.9321e+02
    6      1     11      1   1.1846e+02   4.1179e+02
```

8	9	14	1	1.2210e+02	4.1639e+02
9	8	13	1	1.2566e+02	3.9321e+02
10	9	14	1	1.2538e+02	4.1949e+02
1	2	3	1	1.2271e+02	5.9580e+02
1	6	5	1	1.1186e+02	5.8735e+02
1	6	7	1	1.2203e+02	6.3455e+02
2	1	6	1	1.2419e+02	5.4593e+02
2	3	4	1	1.0563e+02	5.9488e+02
3	4	10	1	1.2558e+02	5.8132e+02
3	4	5	1	1.1168e+02	6.0275e+02
4	10	9	1	1.0563e+02	5.9488e+02
4	5	8	1	1.0942e+02	6.1011e+02
4	5	6	1	1.2151e+02	5.4584e+02
5	4	10	1	1.1168e+02	6.0275e+02
5	8	9	1	1.0990e+02	5.7689e+02
5	6	7	1	1.2571e+02	5.7664e+02
6	5	8	1	1.1411e+02	5.8565e+02
8	9	10	1	1.1202e+02	6.2576e+02

[dihedrals]

;i j k l funcC0 ... C5

11	1	2	3	3	13.80720	0.00000	-13.80720	0.00000	0.00000	0.00000	;
4	3	2	12	3	39.74800	0.00000	-39.74800	0.00000	0.00000	0.00000	;
4	10	9	14	3	39.74800	0.00000	-39.74800	0.00000	0.00000	0.00000	;
4	5	8	13	3	14.22560	0.00000	-14.22560	0.00000	0.00000	0.00000	;
5	8	9	14	3	14.22560	0.00000	-14.22560	0.00000	0.00000	0.00000	;
11	1	6	5	3	20.92000	0.00000	-20.92000	0.00000	0.00000	0.00000	;
6	1	2	12	3	13.80720	0.00000	-13.80720	0.00000	0.00000	0.00000	;
6	5	8	13	3	14.22560	0.00000	-14.22560	0.00000	0.00000	0.00000	;
11	1	6	7	3	29.28800	-8.36800	-20.92000	0.00000	0.00000	0.00000	;
10	9	8	13	3	14.22560	0.00000	-14.22560	0.00000	0.00000	0.00000	;
11	1	2	12	3	13.80720	0.00000	-13.80720	0.00000	0.00000	0.00000	;
13	8	9	14	3	14.22560	0.00000	-14.22560	0.00000	0.00000	0.00000	;
11	1	2	6	3	9.20480	0.00000	-9.20480	0.00000	0.00000	0.00000	;
12	1	2	3	3	9.20480	0.00000	-9.20480	0.00000	0.00000	0.00000	;
5	9	8	13	3	9.20480	0.00000	-9.20480	0.00000	0.00000	0.00000	;
14	8	9	10	3	9.20480	0.00000	-9.20480	0.00000	0.00000	0.00000	;
1	2	3	4	3	39.74800	0.00000	-39.74800	0.00000	0.00000	0.00000	;
1	6	5	4	3	24.05800	0.00000	-24.05800	0.00000	0.00000	0.00000	;
1	6	5	8	3	24.05800	0.00000	-24.05800	0.00000	0.00000	0.00000	;

```
    2     1     6     5     3     20.92000     0.00000    -20.92000     0.00000     0.00000     0.00000   ;
    2     1     6     7     3     20.92000     0.00000    -20.92000     0.00000     0.00000     0.00000   ;
    2     3     4    10     3     39.74800     0.00000    -39.74800     0.00000     0.00000     0.00000   ;
    2     3     4     5     3     39.74800     0.00000    -39.74800     0.00000     0.00000     0.00000   ;
    6     1     2     3     3     13.80720     0.00000    -13.80720     0.00000     0.00000     0.00000   ;
    3     4    10     9     3     39.74800     0.00000    -39.74800     0.00000     0.00000     0.00000   ;
    3     4     5     8     3     33.47200     0.00000    -33.47200     0.00000     0.00000     0.00000   ;
    3     4     5     6     3     33.47200     0.00000    -33.47200     0.00000     0.00000     0.00000   ;
    4    10     9     8     3     39.74800     0.00000    -39.74800     0.00000     0.00000     0.00000   ;
    4     5     8     9     3     14.22560     0.00000    -14.22560     0.00000     0.00000     0.00000   ;
    4     5     6     7     3     24.05800     0.00000    -24.05800     0.00000     0.00000     0.00000   ;
    5     4    10     9     3     39.74800     0.00000    -39.74800     0.00000     0.00000     0.00000   ;
    5     8     9    10     3     14.22560     0.00000    -14.22560     0.00000     0.00000     0.00000   ;
    6     5     4    10     3     33.47200     0.00000    -33.47200     0.00000     0.00000     0.00000   ;
    6     5     8     9     3     14.22560     0.00000    -14.22560     0.00000     0.00000     0.00000   ;
    7     6     5     8     3     24.05800     0.00000    -24.05800     0.00000     0.00000     0.00000   ;
    8     5     4    10     3     33.47200     0.00000    -33.47200     0.00000     0.00000     0.00000   ;
    5     3     4    10     3      9.20480     0.00000     -9.20480     0.00000     0.00000     0.00000   ;
    6     4     5     8     3      9.20480     0.00000     -9.20480     0.00000     0.00000     0.00000   ;
    5     1     6     7     3     87.86400     0.00000    -87.86400     0.00000     0.00000     0.00000   ;

; Include Position restraint file
#ifdef POSRES
#include "posre.itp"
#endif

; Include water topology
#include "amber99sb-ildn.ff/tip3p.itp"

#ifdef POSRES_WATER
; Position restraint for each water oxygen
[ position_restraints ]
;    i funct        fcx          fcy          fcz
     1    1         1000         1000         1000
#endif

; Include topology for ions
#include "amber99sb-ildn.ff/ions.itp"
```

[system]
14 system in water

[molecules]
; Compound nmols
HPA 1
SOL 689
NA 2
CL 2

File S4. Force field parameters of 6AP for Gromacs:

; lig.top created by rdparm2gmx.pl 2018 年 02 月 09 日 星期五 06:28:23 CST

; Include forcefield parameters
#include "amber99sb-ildn.ff/forcefield.itp"

;[defaults]

; nbfunc	comb-rule	gen-pairs	fudgeLJ	fudgeQQ
;1	2	yes	0.5	0.8333

[atomtypes]

;name	bond_type	mass	charge	ptype	sigma	epsilon
nd	nd	0.0000	0.0000	A	3.25000e-01	7.11280e-01
cc	cc	0.0000	0.0000	A	3.39967e-01	3.59824e-01
nh	nh	0.0000	0.0000	A	3.25000e-01	7.11280e-01
hn	hn	0.0000	0.0000	A	1.06908e-01	6.56888e-02
nb	nb	0.0000	0.0000	A	3.25000e-01	7.11280e-01
na	na	0.0000	0.0000	A	3.25000e-01	7.11280e-01
ca	ca	0.0000	0.0000	A	3.39967e-01	3.59824e-01
h5	h5	0.0000	0.0000	A	2.42146e-01	6.27600e-02

[moleculetype]
; Name nrexcl
6AP 3

```
[ atoms ]
;    nr         type  resnr residue  atom   cgnr     charge       mass  typeB    chargeB
      1           na      1    6AP     N9      1   -0.38800  14.000000
      2           cc      1    6AP     C8      2    0.38770  12.000000
      3           nd      1    6AP     N7      3   -0.53310  14.000000
      4           ca      1    6AP     C5      4   -0.27020  12.000000
      5           ca      1    6AP     C4      5    0.49280  12.000000
      6           nb      1    6AP     N3      6   -0.79500  14.000000
      7           ca      1    6AP     C2      7    0.91990  12.000000
      8           nh      1    6AP     N2      8   -0.92650  14.000000
      9           nb      1    6AP     N1      9   -0.84300  14.000000
     10           ca      1    6AP     C6     10    0.78920  12.000000
     11           nh      1    6AP     N6     11   -0.95750  14.000000
     12           hn      1    6AP     H1     12    0.31570   1.000000
     13           h5      1    6AP     H2     13    0.07080   1.000000
     14           hn      1    6AP     H3     14    0.42480   1.000000
     15           hn      1    6AP     H4     15    0.42480   1.000000
     16           hn      1    6AP     H5     16    0.44380   1.000000
     17           hn      1    6AP     H6     17    0.44380   1.000000

[ bonds ]
;   ai     aj funct   r   k
     1     12     1  1.0110e-01  3.4024e+05
     2     13     1  1.0790e-01  2.9790e+05
     8     14     1  1.0140e-01  3.3572e+05
     8     15     1  1.0140e-01  3.3572e+05
    11     16     1  1.0140e-01  3.3572e+05
    11     17     1  1.0140e-01  3.3572e+05
     1      5     1  1.3500e-01  3.9355e+05
     1      2     1  1.3710e-01  3.6719e+05
     2      3     1  1.3350e-01  4.1388e+05
     3      4     1  1.3360e-01  4.1246e+05
     4      5     1  1.3870e-01  4.0033e+05
     4     10     1  1.3870e-01  4.0033e+05
     5      6     1  1.3420e-01  4.0426e+05
     6      7     1  1.3420e-01  4.0426e+05
     7      8     1  1.3640e-01  3.7572e+05
     7      9     1  1.3420e-01  4.0426e+05
     9     10     1  1.3420e-01  4.0426e+05
    10     11     1  1.3640e-01  3.7572e+05
```

```
[ pairs ]
;    ai        aj funct
        12          3           1
         4         13           1
        12          4           1
         4         16           1
         4         17           1
         5         13           1
        12          6           1
         6         14           1
         6         15           1
         9         14           1
         9         15           1
         9         16           1
         9         17           1
        12         13           1
         1         10           1
         1          7           1
         2          6           1
         2         10           1
         3          6           1
         3          9           1
         3         11           1
         4          7           1
         5          9           1
         5         11           1
         5          8           1
         6         10           1
         7         11           1
         8         10           1

[ angles ]
;    ai        aj        ak funct    theta      cth
       1         2        13         1    1.2210e+02    4.1639e+02
       2         1        12         1    1.2566e+02    3.9321e+02
       3         2        13         1    1.2538e+02    4.1949e+02
       5         1        12         1    1.2559e+02    3.9857e+02
       7         8        14         1    1.1613e+02    4.1070e+02
       7         8        15         1    1.1613e+02    4.1070e+02
```

10	11	16	1	1.1613e+02	4.1070e+02
10	11	17	1	1.1613e+02	4.1070e+02
14	8	15	1	1.1485e+02	3.3514e+02
16	11	17	1	1.1485e+02	3.3514e+02
1	5	4	1	1.1834e+02	5.8752e+02
1	5	6	1	1.2707e+02	5.9078e+02
1	2	3	1	1.1202e+02	6.2576e+02
2	1	5	1	1.1315e+02	5.7287e+02
2	3	4	1	1.0547e+02	6.0450e+02
3	4	5	1	1.1972e+02	5.8693e+02
3	4	10	1	1.1972e+02	5.8693e+02
4	5	6	1	1.2263e+02	5.7873e+02
4	10	9	1	1.2263e+02	5.7873e+02
4	10	11	1	1.2013e+02	5.8024e+02
5	4	10	1	1.1997e+02	5.6216e+02
5	6	7	1	1.1586e+02	5.7396e+02
6	7	8	1	1.1695e+02	6.1254e+02
6	7	9	1	1.2719e+02	5.9229e+02
7	9	10	1	1.1586e+02	5.7396e+02
8	7	9	1	1.1695e+02	6.1254e+02
9	10	11	1	1.1695e+02	6.1254e+02

[dihedrals]

;i j k l funcC0 ... C5

12	1	2	3	3	14.22560	0.00000	-14.22560	0.00000	0.00000	0.00000	;
4	3	2	13	3	39.74800	0.00000	-39.74800	0.00000	0.00000	0.00000	;
12	1	5	4	3	2.51040	0.00000	-2.51040	0.00000	0.00000	0.00000	;
4	10	11	16	3	8.78640	0.00000	-8.78640	0.00000	0.00000	0.00000	;
4	10	11	17	3	8.78640	0.00000	-8.78640	0.00000	0.00000	0.00000	;
5	1	2	13	3	14.22560	0.00000	-14.22560	0.00000	0.00000	0.00000	;
12	1	5	6	3	2.51040	0.00000	-2.51040	0.00000	0.00000	0.00000	;
6	7	8	14	3	8.78640	0.00000	-8.78640	0.00000	0.00000	0.00000	;
6	7	8	15	3	8.78640	0.00000	-8.78640	0.00000	0.00000	0.00000	;
9	7	8	14	3	8.78640	0.00000	-8.78640	0.00000	0.00000	0.00000	;
9	7	8	15	3	8.78640	0.00000	-8.78640	0.00000	0.00000	0.00000	;
9	10	11	16	3	8.78640	0.00000	-8.78640	0.00000	0.00000	0.00000	;
9	10	11	17	3	8.78640	0.00000	-8.78640	0.00000	0.00000	0.00000	;
12	1	2	13	3	14.22560	0.00000	-14.22560	0.00000	0.00000	0.00000	;
12	1	2	5	3	9.20480	0.00000	-9.20480	0.00000	0.00000	0.00000	;
13	1	2	3	3	9.20480	0.00000	-9.20480	0.00000	0.00000	0.00000	;

```
7	14	8	15	3	9.20480	0.00000	-9.20480	0.00000	0.00000	0.00000	;
10	16	11	17	3	9.20480	0.00000	-9.20480	0.00000	0.00000	0.00000	;
1	5	4	3	3	30.33400	0.00000	-30.33400	0.00000	0.00000	0.00000	;
1	5	4	10	3	30.33400	0.00000	-30.33400	0.00000	0.00000	0.00000	;
1	5	6	7	3	40.16640	0.00000	-40.16640	0.00000	0.00000	0.00000	;
1	2	3	4	3	39.74800	0.00000	-39.74800	0.00000	0.00000	0.00000	;
2	1	5	4	3	2.51040	0.00000	-2.51040	0.00000	0.00000	0.00000	;
2	1	5	6	3	2.51040	0.00000	-2.51040	0.00000	0.00000	0.00000	;
2	3	4	5	3	40.16640	0.00000	-40.16640	0.00000	0.00000	0.00000	;
2	3	4	10	3	40.16640	0.00000	-40.16640	0.00000	0.00000	0.00000	;
5	1	2	3	3	14.22560	0.00000	-14.22560	0.00000	0.00000	0.00000	;
3	4	5	6	3	30.33400	0.00000	-30.33400	0.00000	0.00000	0.00000	;
3	4	10	9	3	30.33400	0.00000	-30.33400	0.00000	0.00000	0.00000	;
3	4	10	11	3	30.33400	0.00000	-30.33400	0.00000	0.00000	0.00000	;
4	5	6	7	3	40.16640	0.00000	-40.16640	0.00000	0.00000	0.00000	;
4	10	9	7	3	40.16640	0.00000	-40.16640	0.00000	0.00000	0.00000	;
5	4	10	9	3	30.33400	0.00000	-30.33400	0.00000	0.00000	0.00000	;
5	4	10	11	3	30.33400	0.00000	-30.33400	0.00000	0.00000	0.00000	;
5	6	7	8	3	40.16640	0.00000	-40.16640	0.00000	0.00000	0.00000	;
5	6	7	9	3	40.16640	0.00000	-40.16640	0.00000	0.00000	0.00000	;
6	5	4	10	3	30.33400	0.00000	-30.33400	0.00000	0.00000	0.00000	;
6	7	9	10	3	40.16640	0.00000	-40.16640	0.00000	0.00000	0.00000	;
7	9	10	11	3	40.16640	0.00000	-40.16640	0.00000	0.00000	0.00000	;
8	7	9	10	3	40.16640	0.00000	-40.16640	0.00000	0.00000	0.00000	;
5	10	4	3	3	9.20480	0.00000	-9.20480	0.00000	0.00000	0.00000	;
4	1	5	6	3	9.20480	0.00000	-9.20480	0.00000	0.00000	0.00000	;
6	9	7	8	3	9.20480	0.00000	-9.20480	0.00000	0.00000	0.00000	;
4	9	10	11	3	9.20480	0.00000	-9.20480	0.00000	0.00000	0.00000	;

; Include Position restraint file
#ifdef POSRES
#include "posre.itp"
#endif

; Include water topology
#include "amber99sb-ildn.ff/tip3p.itp"

#ifdef POSRES_WATER
; Position restraint for each water oxygen
[ position_restraints ]
```

```
;    i funct              fcx                fcy                fcz
     1       1              1000              1000              1000
#endif

; Include topology for ions
#include "amber99sb-ildn.ff/ions.itp"

[ system ]
17 system in water

[ molecules ]
; Compound                nmols
6AP                          1
SOL                 776
NA                          2
CL                          2
```

Table S1. The parameters of the simulated systems produced by the Leap module in Amber[a]

Ligands	RNA	Box size (Å)	Atom number of system	Counter ions(Na+)
HPA	WT	92.736×70.287×60.137	34937	66
	A24U	89.960×70.742×59.445	33536	66
	U25A/A46G	90.120×71.1985×9.803	34137	66
	A24U/U25A/A46G	90.026×71.734×59.215	34080	66
6AP	WT	92.736×70.287×60.137	34940	66
	U25A/A46G/C74U	89.894×71.278×58.972	33677	66
	A24U/U25A/A46G/C74U	90.047×72.266×63.302	36566	66

[a]All information were automatically produced by the Leap module in Amber by setting the parameters described in our manuscript.

Table S2. Hydrogen bonding interactions of HPA with key residues in the WT and mutated GRs

[a]Hydrogen bonds	WT GR		A24U		U25A/A46G		A24U/U25A/A46G	
	[b]Occupancy (%)	Distance (Å)	Occupancy (%)	Distance (Å)	Occupancy (%)	Distance (Å)	Occupancy (%)	Distance (Å)
[c]HPA@O6...C74@N4...H41	73.92	2.98	[d] --	--	92.85	2.94	94.46	2.95
HPA@N3...U51@N3...H3	58.24	3.00	84.86	3.01	45.14	3.11	97.58	29.7
HPA@O6...U22@O2′-HO2′	--	--	33.32	2.81	--	--	--	--
U22@O2′...HPA@N7-H3	80.17	2.93	95.44	2.89	91.22	2.91	91.24	2.96
C74@N3...HPA@N1-H1	78.84	2.89	--	--	93.17	2.91	95.17	2.95
C74@O2...HPA@N1-H1	--	--	98.58	2.96	--	--	--	--

[a]Hydrogen bonds are determined by the acceptor···donor distance of < 3.5Å and acceptor···H-donor angle of > 120°.

[b]Occupancy(%) is defined as the percentage of simulation time that a specific hydrogen bond exists.

[c]The full lines indicate chemical bonds, and the dotted lines describe hydrogen bonding interactions.

[d]The symbol "--" indicates that occupancy of hydrogen bonds formed between HPA and nucleotides is lower than 20%.

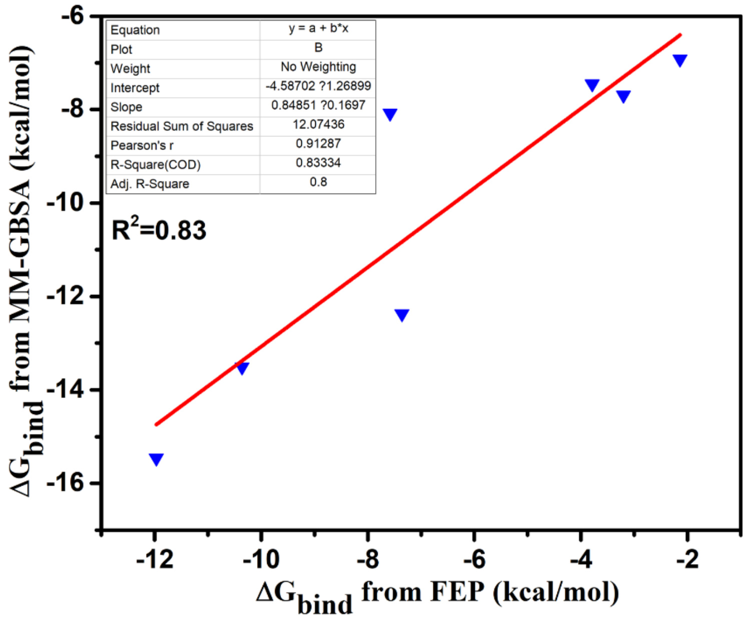

Figure S1. Correlation relationship between binding free energies calculated by FEP method and that by MM-GBSA method.

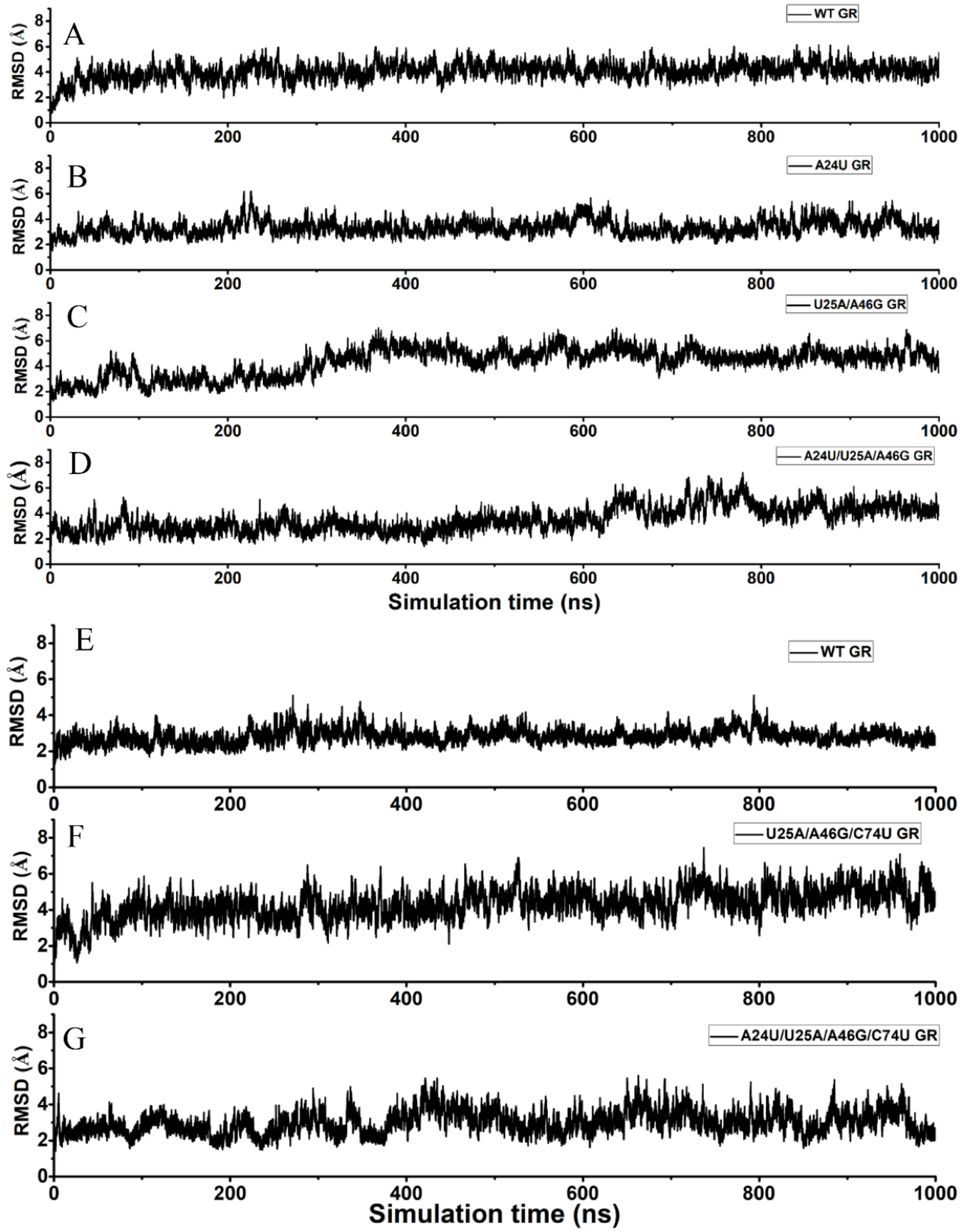

Figure S2. Root-mean-square deviations (RMSDs) of atoms P1, O3′, O5′, C3′, C4′ and C5′ in GR: (A)-(D) the WT and mutated GRs with ligand HPA, (E)-(G) the WT and mutated GRs with ligand 6AP.

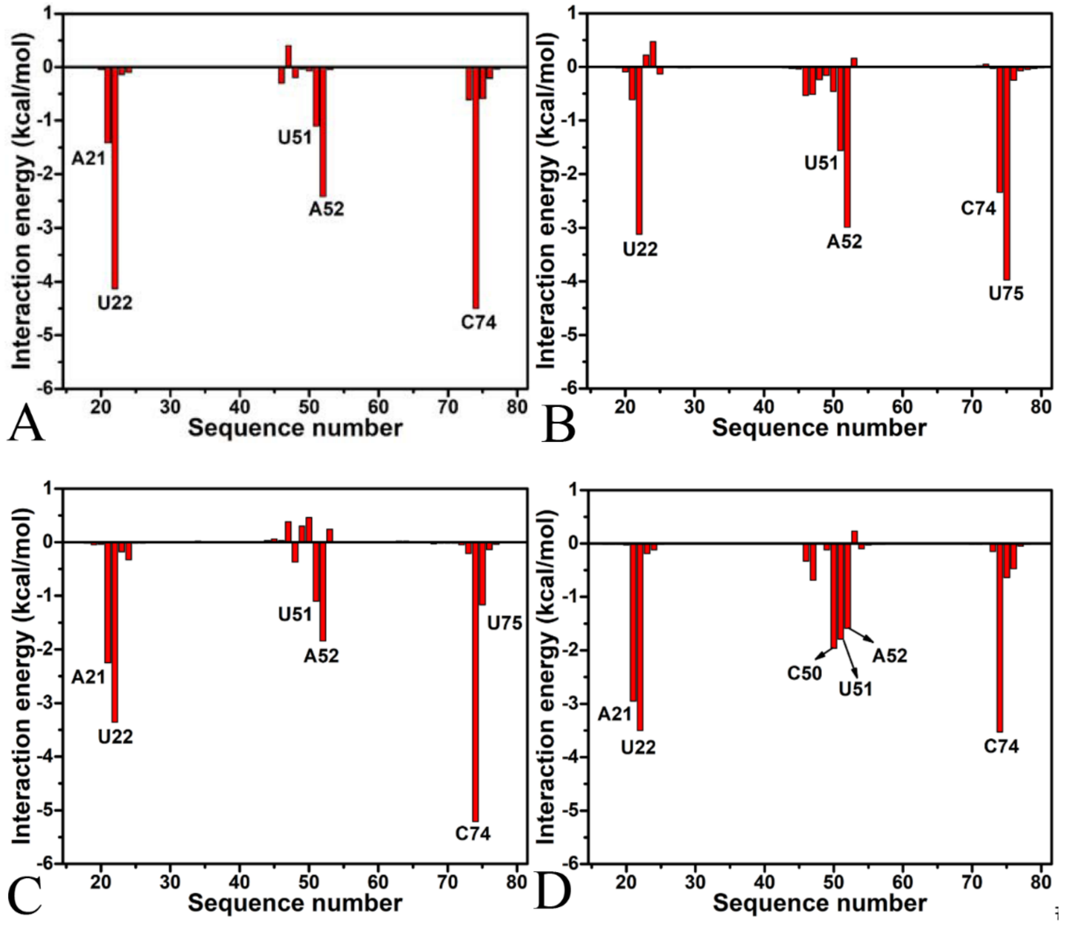

Figure S3. HPA-nucleotide interaction spectrum: (A) the WT GR, (B) the GR with mutation A24U, (C) the GR with mutations U25A/A46G and (D) the GR with mutations A24U/U25A/A46G. Only nucleotides of interaction energies stronger than 1.0 kcal/mol were labeled.

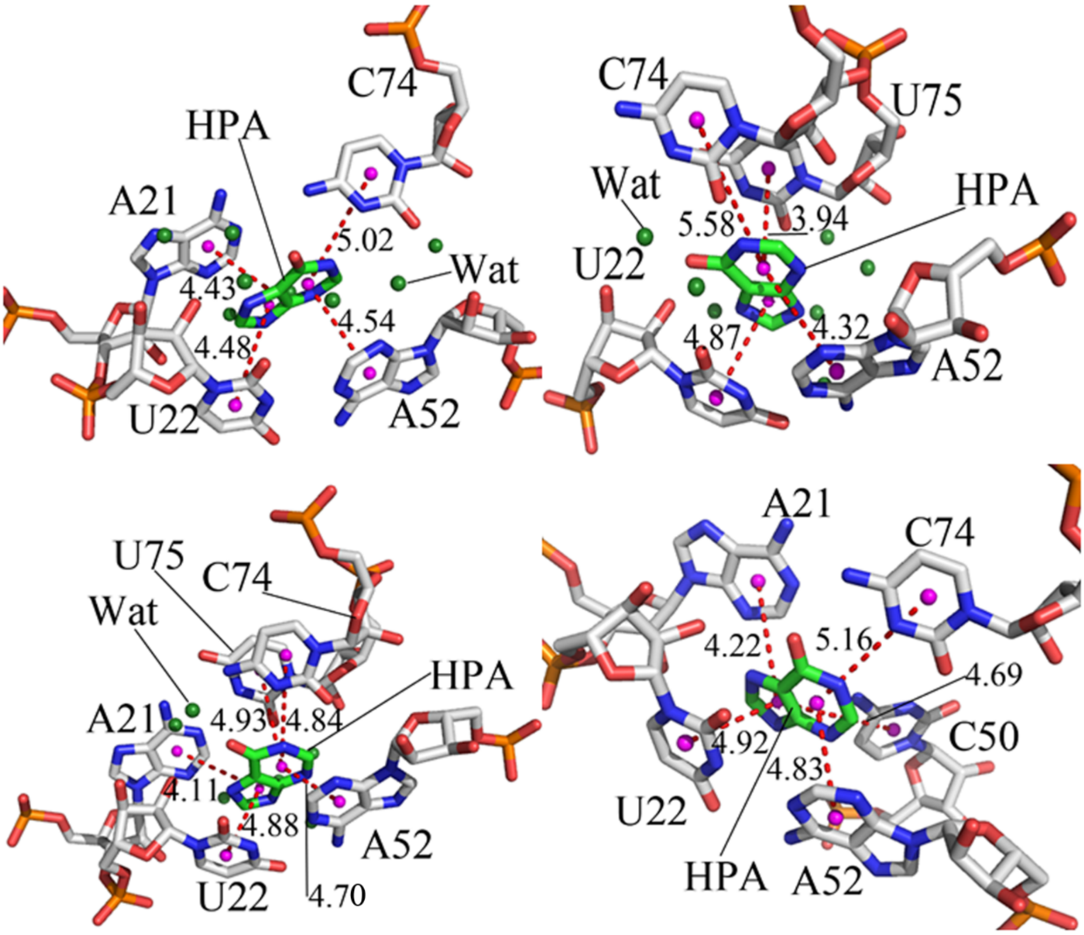

Figure S4. Hydrophobic interactions of HPA with key nucleotides in the WT and mutated GRs: (A) WT, (B) A24U, (C) U25A/A46G and (D) A24U/U25A/A46G. The key nucleotides and HPA are shown in stick modes and water molecules in the forest color balls. The magenta balls represent pseudoatoms forming hydrophobic interactions between 6AP and nucleotides.

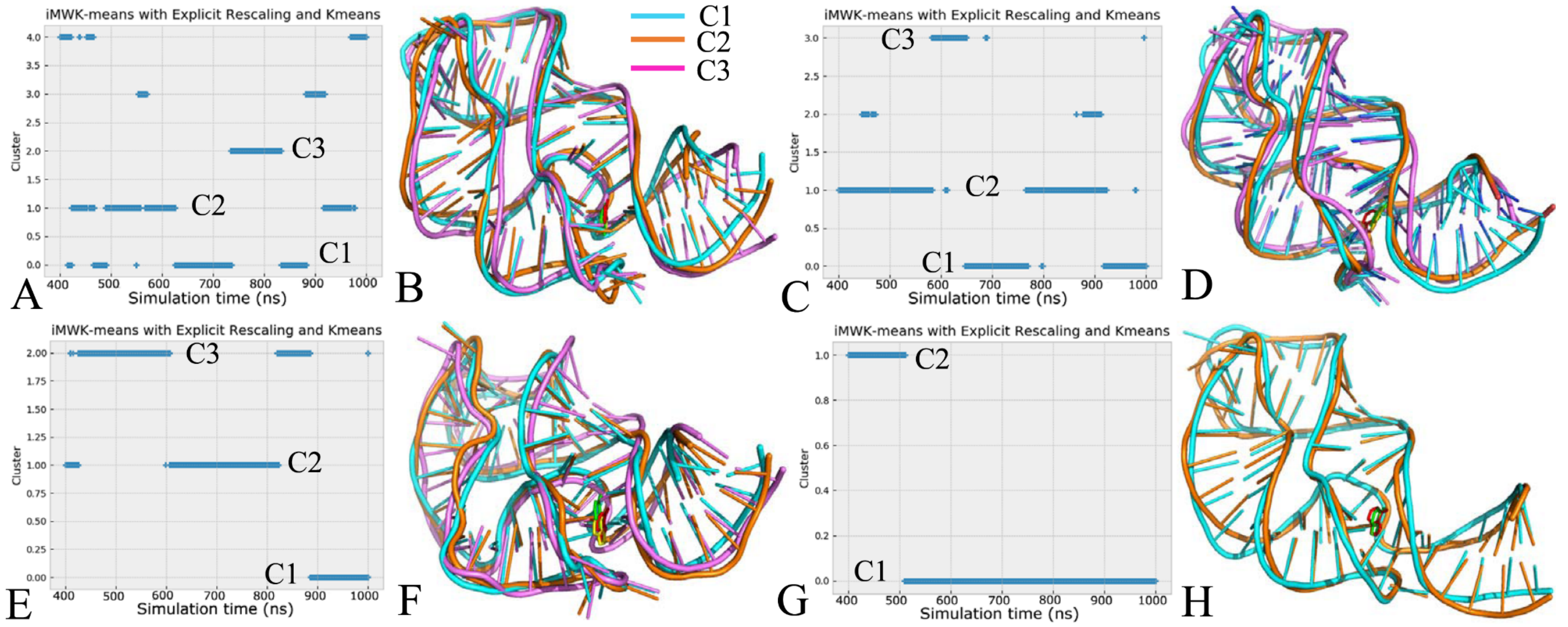

Figure S5. Cluster analyses and superimpositions of corresponding structures: (A), (C), (E) and (G) depict the cluster analyses performed by using iMWK-Means method, and (A), (C), (E) and (G) respectively represent the WT, A24U, U25A/A46G and A24U/U25A/A46G GRs complexed with HPA; (B), (D), (F) and (H) display superimpositions of structures extracted from different clusters C1, C2 and C3. GRs are shown in cartoon modes and HPA is displayed in stick modes with green, red and yellow.

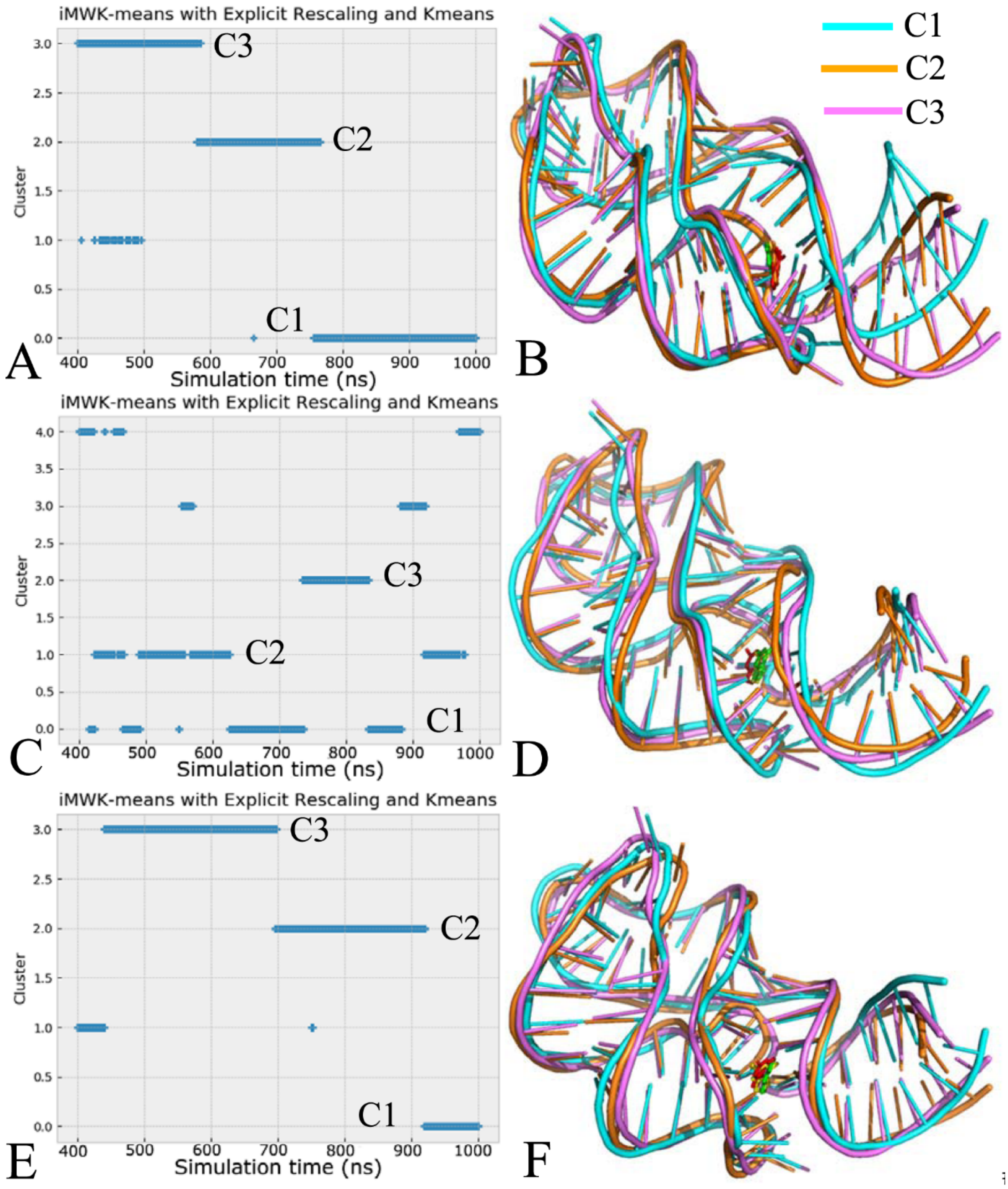

Figure S6. Cluster analyses and superimpositions of corresponding structures: (A), (C) and (E) depict the cluster analyses performed by using iMWK-Means method, and (A), (C) and (E) respectively indicate the WT, U25A/A46G/C74U and A24U/U25A/A46G/C74U GRs complexed with 6AP; (B), (D)and (F) show superimpositions of structures extracted from different clusters C1, C2 and C3. GRs are depicted in cartoon modes and 6AP is shown in stick modes with green, red and yellow.

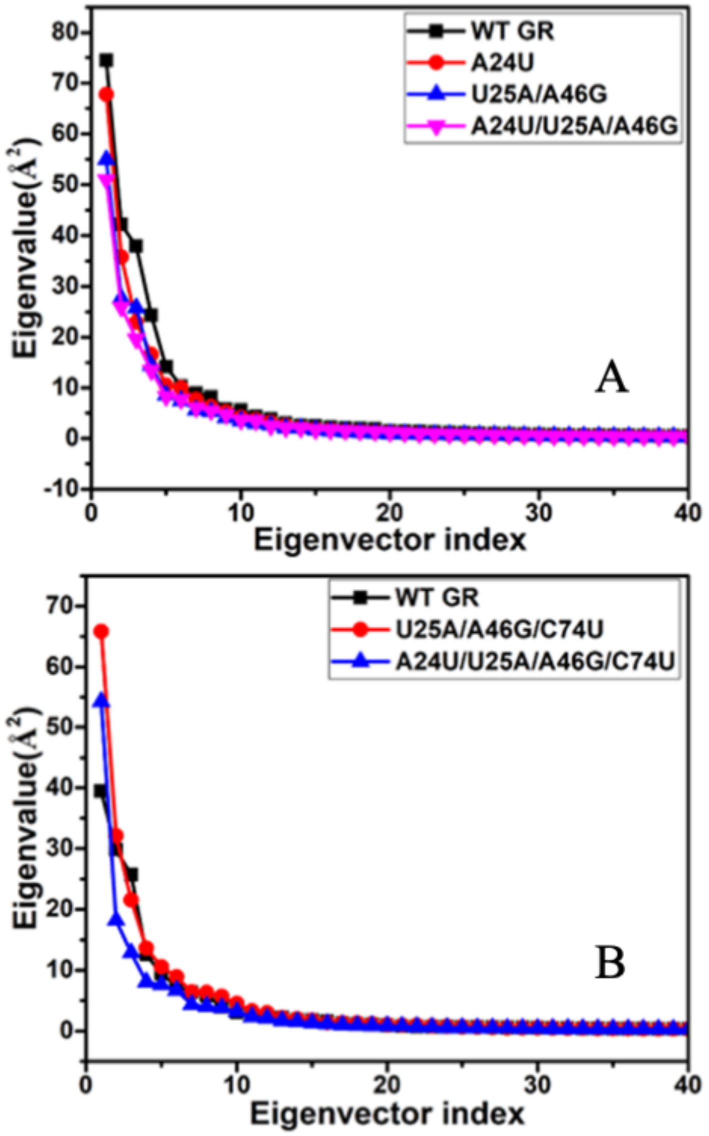

Figure S7. Comparison of the eigenvalues plotted against the corresponding eigenvector indices obtained from the covariance matrix of C1′ atoms in GR constructed from the equilibrated MD trajectories: (A) the WT and mutated GRs complexed with HPA and (B) the WT and mutated GRs complexed with 6AP.

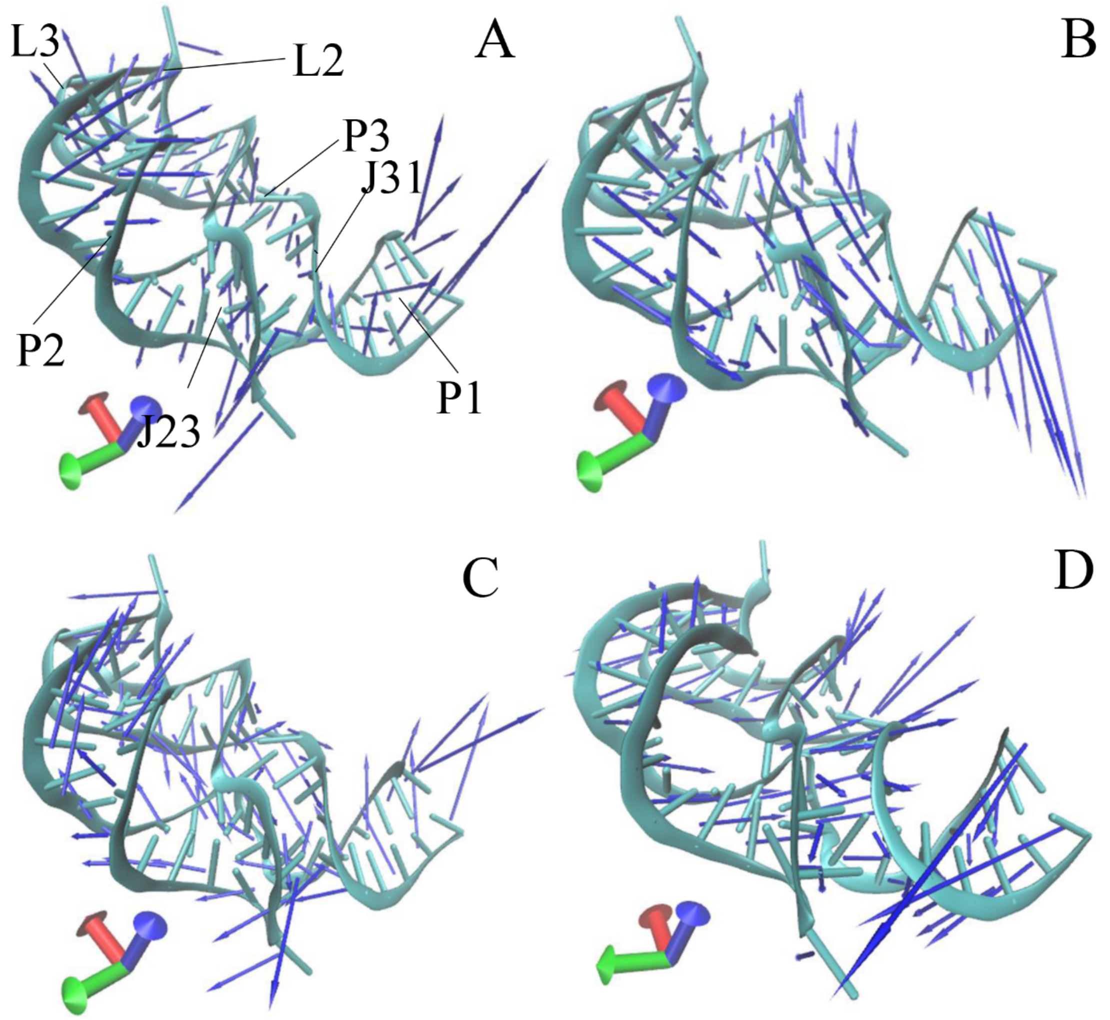

Figure S8. Collective motions reflected by the first eigenvector corresponding to the principal component PC1 obtained by performing principal component analysis (PCA) on the equilibrated MD trajectories of the WT and mutated GRs complexed with HPA: (A) WT, (B) A24U, (C) U25A/A46G and (D) A24U/U25A/A46G. In this figure, the direction and length of the arrows respectively reflect the motion direction and strength of nucleotides in GR.

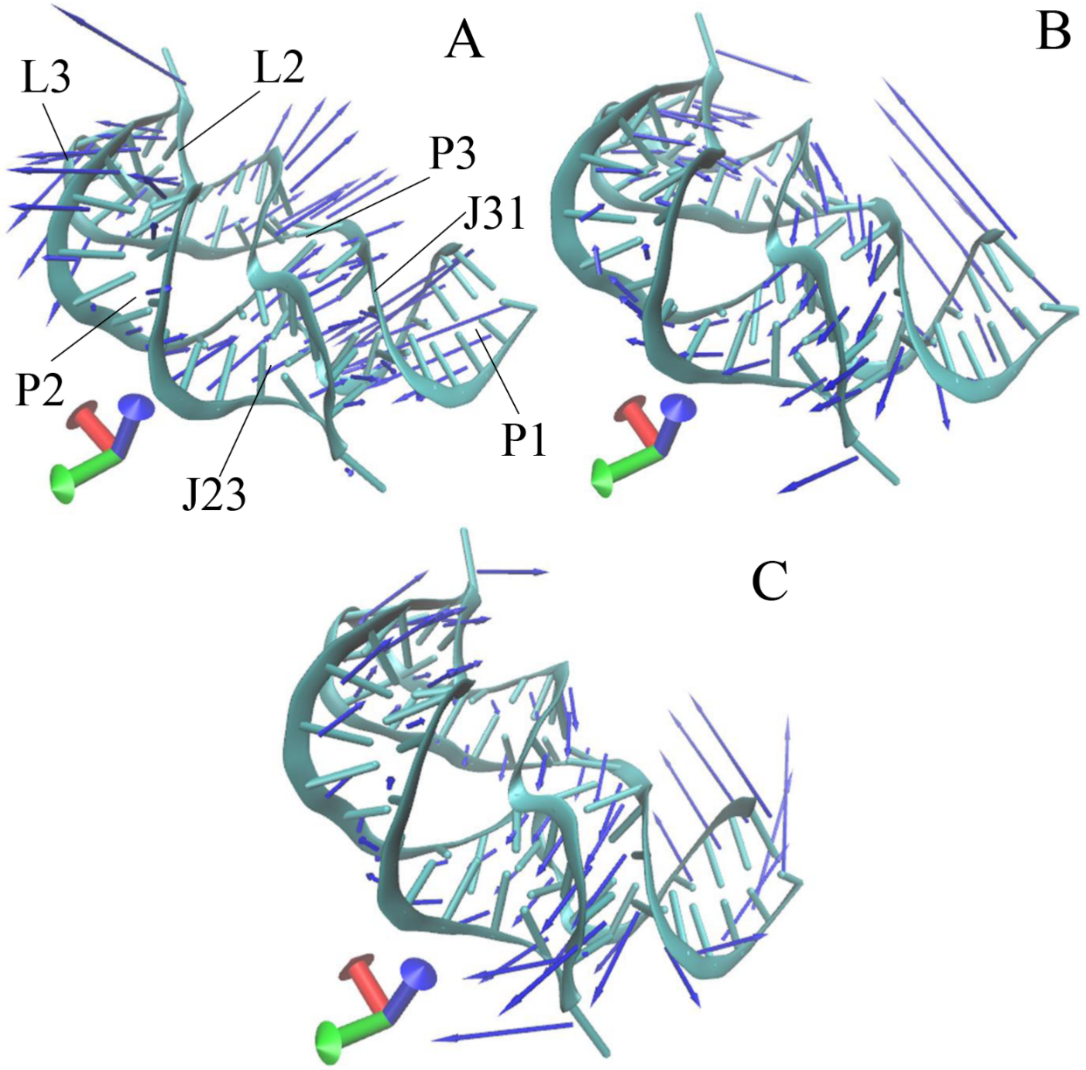

Figure S9. Collective motions reflected by the first eigenvector corresponding to the principal component PC1 obtained by performing principal component analysis (PCA) on the equilibrated MD trajectories of the WT and mutated GRs complexed with 6AP: (A) WT, (B) U25A/A46G/C74U and (C) A24U/U25A/A46G/C74U. In this figure, the direction and length of the arrows respectively reflect the motion direction and strength of nucleotides in GR.

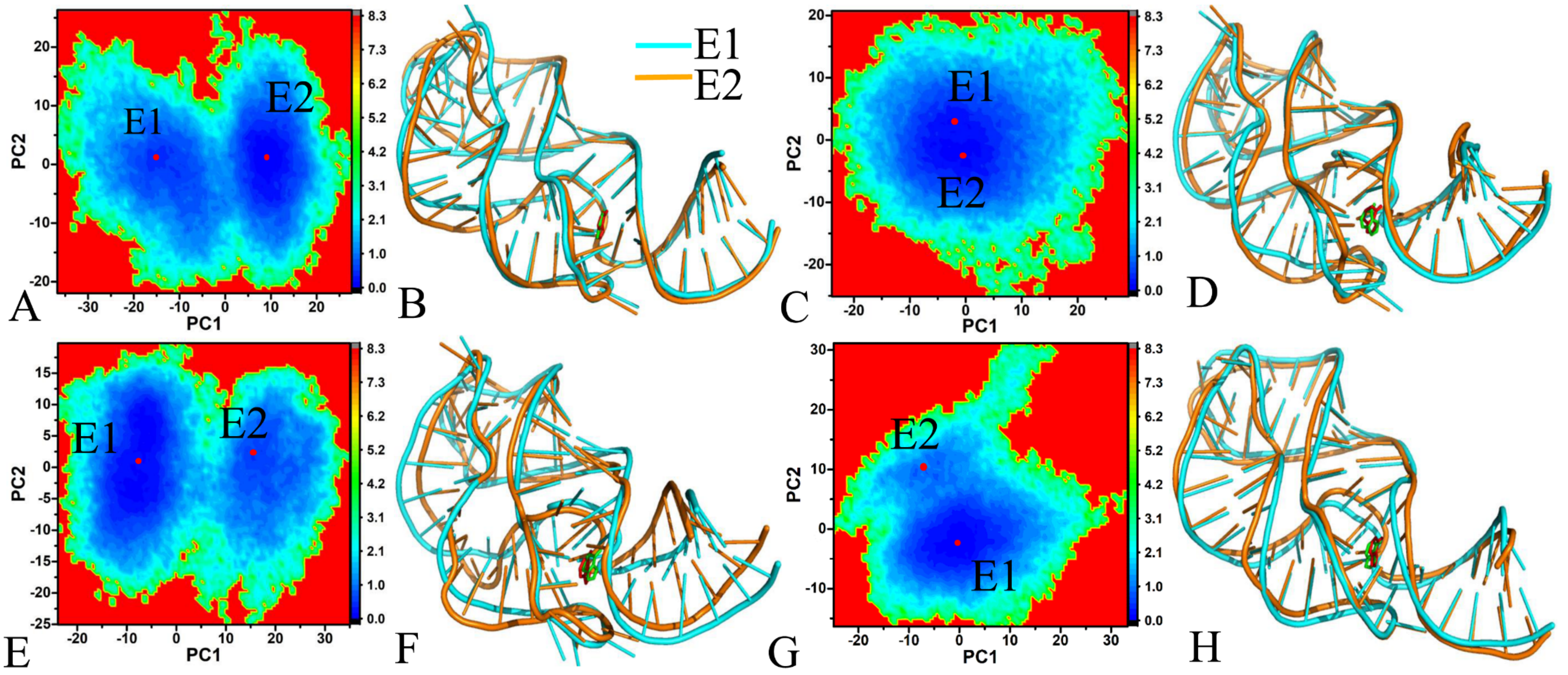

Figure S10. Free energy landscapes and structural superimposition of the WT and mutated GRs with HPA: (A), (C), (E) and (G) depict free energy landscapes constructed using projection of MD trajectories on the first two principal components PC1 and PC2 from the diagonalization of covariance matrix, among which (A), (C), (E) and (G) separately correspond to the WT, A24U, U25A/A46G and A24U/U25A/A46G GRs; (B), (D), (F) and (H) display superimpositions of structures represented by free energy basins E1 and E2, and (B), (D), (F) and (H) respectively indicate the WT, A24U, U25A/A46G and A24U/U25A/A46G GRs. GRs are shown in cartoon modes and HPA is displayed in stick modes with green, red and yellow.

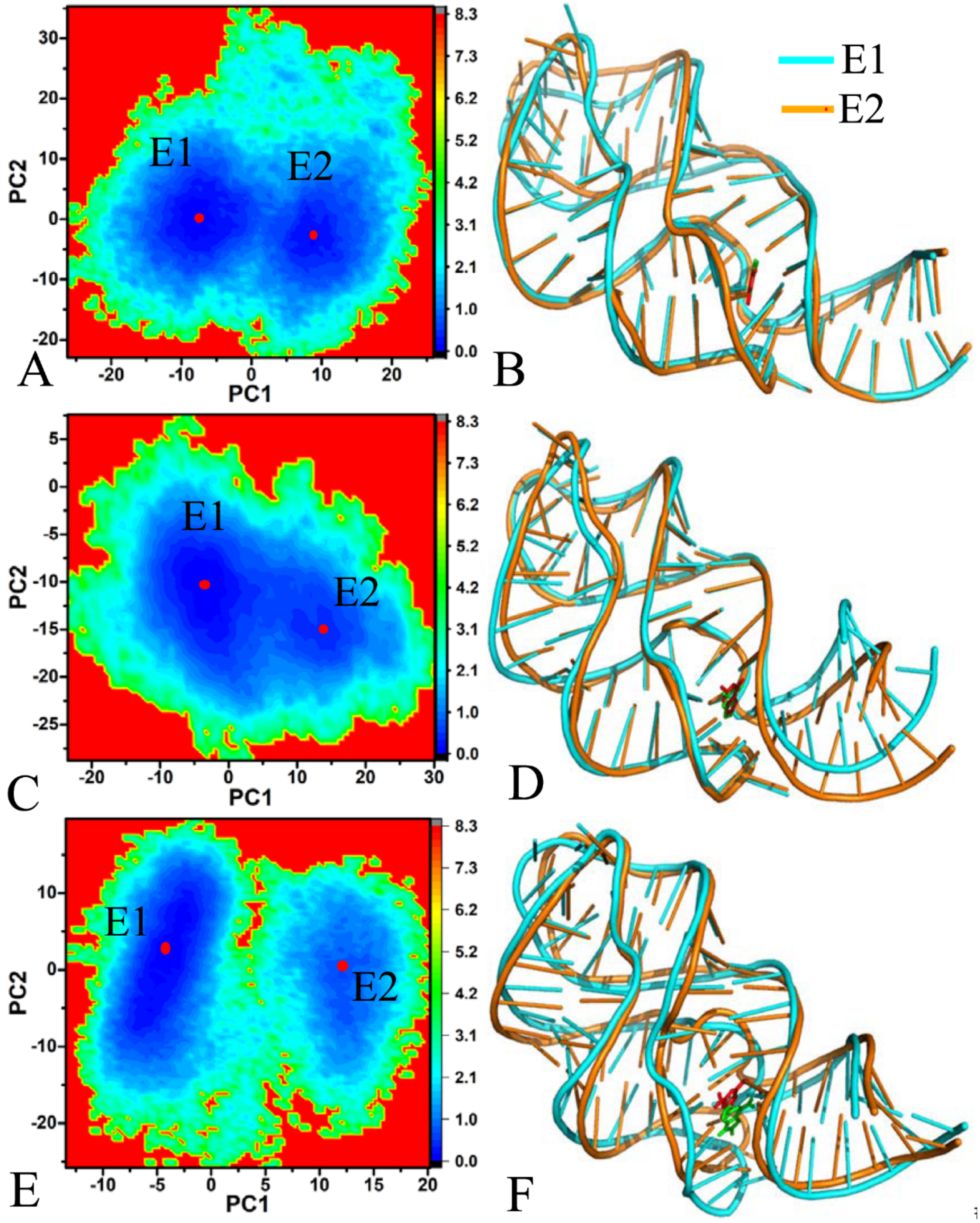

Figure S11. Free energy landscapes and structural superimposition of the WT and mutated GRs with 6AP: (A), (C) and (E) depict free energy landscapes constructed using projection of MD trajectories on the first two principal components PC1 and PC2 from the diagonalization of covariance matrix, among which (A), (C) and (E) separately correspond to the WT, U25A/A46G/C74U and A24U/U25A/A46G/C74U GRs; (B), (D) and (F) display superimpositions of structures represented by free energy basins E1 and E2, and (B), (D) and (F) respectively indicate the WT, U25A/A46G/C74U and A24U/U25A/A46G/C74U GRs. GRs are shown in cartoon modes and 6AP is displayed in stick modes with green, red and yellow
